# A screen for *Plasmodium falciparum* sporozoite surface protein binding to human hepatocyte surface receptors identifies novel host–pathogen interactions

**DOI:** 10.1186/s12936-024-04913-2

**Published:** 2024-05-16

**Authors:** Rameswara R. Segireddy, Hugo Belda, Annie S. P. Yang, Kirsten Dundas, Julia Knoeckel, Francis Galaway, Laura Wood, Doris Quinkert, Ellen Knuepfer, Moritz Treeck, Gavin J. Wright, Alexander D. Douglas

**Affiliations:** 1 Jenner Institute, University of Oxford, Old Road Campus Research Building, Roosevelt Drive, Oxford, OX3 7DQ UK; 2 Signalling in Apicomplexan Parasites Laboratory, The Francis Crick Institute, Midland Road, London, NW1 1AT UK; 3 Research Center for Infectious Diseases, Department of Medical Microbiology, Radboud University Medical Center, Geert Grooteplein Zuid 10, 6525 GA Nijmegen, The Netherlands; 4 Wellcome Sanger Institute, Wellcome Genome Campus, Hinxton, Cambridge, CB10 1SA UK; 5 Department of Biochemistry, University of Oxford, South Parks Rd, Oxford, OX1 3QU UK; 6 The Royal Veterinary College, North Mymms, Hawkshead Lane, Hatfield, Hertfordshire AL9 7TA UK; 7 Department of Biology, Hull York Medical School, York Biomedical Research Institute, University of York, Wentworth Way, York, YO10 5DD UK

**Keywords:** Malaria, *Plasmodium falciparum*, Sporozoite, AVEXIS, Pf34, FGFR4, PfRON6, LDLR, PIESP15

## Abstract

**Background:**

Sporozoite invasion of hepatocytes is an essential step in the *Plasmodium* life-cycle and has similarities, at the cellular level, to merozoite invasion of erythrocytes. In the case of the *Plasmodium* blood-stage, efforts to identify host–pathogen protein–protein interactions have yielded important insights including vaccine candidates. In the case of sporozoite-hepatocyte invasion, the host–pathogen protein–protein interactions involved are poorly understood.

**Methods:**

To gain a better understanding of the protein–protein interaction between the sporozoite ligands and host receptors, a systematic screen was performed. The previous *Plasmodium falciparum* and human surface protein ectodomain libraries were substantially extended, resulting in the creation of new libraries comprising 88 *P. falciparum* sporozoite protein coding sequences and 182 sequences encoding human hepatocyte surface proteins. Having expressed recombinant proteins from these sequences, a plate-based assay was used, capable of detecting low affinity interactions between recombinant proteins, modified for enhanced throughput, to screen the proteins for interactions. The novel interactions identified in the screen were characterized biochemically, and their essential role in parasite invasion was further elucidated using antibodies and genetically manipulated *Plasmodium* parasites.

**Results:**

A total of 7540 sporozoite-hepatocyte protein pairs were tested under conditions capable of detecting interactions of at least 1.2 µM *K*_D_. An interaction between the human fibroblast growth factor receptor 4 (FGFR4) and the *P. falciparum* protein Pf34 is identified and reported here, characterizing its affinity and demonstrating the blockade of the interaction by reagents, including a monoclonal antibody. Furthermore, further interactions between Pf34 and a second *P. falciparum* rhoptry neck protein, PfRON6, and between human low-density lipoprotein receptor (LDLR) and the *P. falciparum* protein PIESP15 are identified.

Conditional genetic deletion confirmed the essentiality of PfRON6 in the blood-stage, consistent with the important role of this protein in parasite lifecycle. Pf34 was refractory to attempted genetic modification. Antibodies to Pf34 abrogated the interaction and had a modest effect upon sporozoite invasion into primary human hepatocytes.

**Conclusion:**

Pf34 and PfRON6 may be members of a functionally important invasion complex which could be a target for future interventions. The modified interaction screening assay, protein expression libraries and *P. falciparum* mutant parasites reported here may be a useful tool for protein interaction discovery and antigen candidate screening which could be of wider value to the scientific community.

**Supplementary Information:**

The online version contains supplementary material available at 10.1186/s12936-024-04913-2.

## Background

Malaria is a mosquito-borne infectious disease caused by protozoan parasites of the genus *Plasmodium.* Despite some progress in malaria control across the globe, 241 million cases still occurred worldwide in 2020, causing 627,000 deaths, mostly young children in Africa [1]. *Plasmodium falciparum* is the main disease-causing species and has a complex life cycle, shuttling between humans and the *Anopheles* mosquito vector. Human infection is initiated when the mosquito releases sporozoites into the skin while obtaining a blood meal. The deposited sporozoites glide through the skin, enter the bloodstream and traffic to the liver. In the liver, sporozoites actively migrate through several hepatocytes prior to productive invasion, which is characterized by the formation of a specialized compartment, the parasitophorous vacuole (PV). Within the PV, sporozoites undergo several rounds of asexual replication producing thousands of merozoites. Upon rupture of infected hepatocytes, merozoites are released into the blood, invade and replicate inside erythrocytes, leading to the symptoms and complications of malaria [2].

Sporozoite invasion of the hepatocyte is an obligatory step in this life cycle. The *Plasmodium* spp. sporozoite and merozoite share a repertoire of subcellular organelles with each other and with the extracellular stages of other apicomplexan parasites, such as *Toxoplasma gondii*, reflecting their shared specialization in the invasion of host cells. A stepwise invasion process appears to be conserved across these parasites, comprising initial low-affinity attachment, calcium-signalling-dependent release of parasite adhesins capable of binding host proteins, formation of a moving junction, and finally actin/myosin-dependent motile invasion into a parasitophorous vacuole [3]. Several parasite ligand—host receptor interactions have been implicated in *P. falciparum* merozoite—human erythrocyte invasion, and blockade of one of these (the interaction of *P. falciparum* reticulocyte binding protein homologue 5 [PfRH5] with basigin) is now a leading vaccine strategy [4]. A number of host and pathogen proteins have been implicated in sporozoite-hepatocyte invasion, as recently reviewed by some of the current authors [5, 6]. Despite this, it is striking that no specific host–pathogen protein–protein interactions have yet been shown to be essential for this process. To our knowledge, no parasite proteins have been shown to interact with putative hepatocyte receptors such as CD81 and scavenger receptor B1 (SR-BI) [7–10], no host proteins have been shown to interact with putative parasite invasins such as circumsporozoite protein (CSP), P36 and P52 [10–13], and no clear role in hepatocyte invasion has been shown for the interaction of *P. falciparum* thrombospondin-related anonymous protein (TRAP) with integrin α_v_β_3_ [5]. An interaction between host Ephrin type-A receptor 2 (EphA2) and parasite P36 has been suggested but not biochemically demonstrated, and EphA2 has subsequently been shown not to be required for sporozoite invasion [13, 14].

It was hypothesized that, like merozoite-erythrocyte invasion, sporozoite-hepatocyte invasion involves multiple protein–protein interactions, identification of which would enable improved vaccine strategies. Biologically important extracellular protein–protein interactions are often of low affinity and can be very transient (for example, the PfRH5-basigin interaction has 1.2 μM affinity and a half-time of 2.7 s [15]). A plate-based assay, termed the AVidity-based EXtracellular Interaction Screen (AVEXIS) [16], used for identifying such interactions, was previously reported by some of co-authors of this study. Therefore, an AVEXIS was performed to identify sporozoite-hepatocyte protein–protein interactions, complementing a recently-published study of interactions between *P. falciparum* sporozoite proteins themselves [17].

## Methods

### Ethics statement

Polyclonal antisera were raised against Pf34 and FGFR4 by injecting purified proteins into rabbits. Rabbit immunization was performed by Cambridge Research Biochemicals (Billingham, UK) in accordance with the U.K. Animals (Scientific Procedures) Act 1986 (ASPA). Approvals were obtained from an animal ethics committee for the animal studies carried out in this study. Briefly, 2 X 20 week old male out-bred New Zealand rabbits were immunized with 100 μg protein on days 0, 14, 28, 42 and 56. The first immunization was performed subcutaneously nodal with 100 μg protein in Freund’s Complete adjuvant. All following immunizations were performed subcutaneously dorsal with 100 μg protein Freund’s Incomplete adjuvant. On day 62, the rabbits were given a Medetomidine & Buprenorphine intravenously as premedication. Once sedated, they were then anaesthetized with Isoflurane. Once anaesthetized, the rabbits were then exsanguinated by cardiac puncture and harvested the blood. Upon completion of exsanguination, an overdose of Pentobarbitone is also administered. Blood was stored at 4 °C till clotted, then centrifuged at 2000 RPM. Serum was separated, aliquoted and stored in − 20 °C.

Primary human liver cells were freshly isolated from remnant surgical material. The samples were anonymized, and general approval for use of remnant surgical material with informed consent for research use was granted in accordance to the Dutch ethical legislation as described in the Medical Research (Human Subjects) Act and confirmed by the Committee on Research involving Human Subjects, in the region of Arnhem-Nijmegen, the Netherlands. Human RBCs were acquired from healthy donors (National Health Service Blood and Transplant (NHSBT) service, Colindale, London, UK) with informed consent for research use in accordance with the UK Human Tissue Act and ethics approval was obtained for the use of the blood samples.

### Design of sporozoite and hepatocyte surface protein libraries

Comprehensive lists of sporozoite and hepatocyte surface protein constructs used in this study are provided in Additional file 2: Table S1 and Additional file 3: Table S2, respectively.

*Plasmodium falciparum* sporozoite surface proteins were selected for study on the basis of their estimated abundance from available surface proteomic and transcriptomic data, plus review of published functional data. In brief, a list of all *P. falciparum* proteins including predicted signal peptides or transmembrane domains was downloaded from PlasmoDB (accessed October 2016). The selection of proteins for manual review was subsequently carried out using broad criteria. Proteins were considered further if they were among the top 30% of those on the list by abundance in a mass spectrometric analysis of the sporozoite surface proteome [18] or the same authors’ re-analysis of a previous whole-sporozoite proteome [19], or the original report of the sporozoite proteome [20]. Given the limited sensitivity of mass spectrometry for certain proteins, the inclusion criteria comprised the top 5% of proteins on the list, with transcripts exhibiting the highest abundance in two profiles of sporozoite RNA [21] and RNA-seq data (provided by Hoffman et al. to PlasmoDB.org). Consideration was also given to lists of proteins previously found to be present in the micronemes and rhoptries of other *Plasmodium* spp. life-cycle stages [22, 23], and genes for disruption resulted in sporozoite or liver-stage phenotypes, as reported in the RMgmDB transgenic parasite database [24]. These lists were then manually synthesized and reviewed, together with annotation information in PlasmoDB, to compile a set of 79 proteins for which there was reasonable evidence of presence on the surface of the sporozoite (or release to the surface from intracellular organelles during invasion). A further four proteins were added based upon published functional information suggesting a role in sporozoite-hepatocyte attachment and/or invasion (LIMP [25], MB2 [26], LSA-3 [27] and STARP [28]). PfRH5 was included on the basis of its known role in the blood-stage, despite the absence of any known evidence indicating its function in the sporozoite. The total number of proteins selected for study was thus 84 (Additional file 1: Fig. S1). Plasmids encoding the ectodomains of 45 of these (though with different tags from our bait configuration) were already available in a previously designed library [17].

For the remaining sporozoite proteins, starting with predicted amino acid sequences from PlasmoDB, SignalP 4.1 and TMHMM v2.0 web servers were used to identify signal peptide and transmembrane domains and hence identify ectodomains. Endogenous signal peptides were replaced with a mammalian signal peptide (from mouse immunoglobulin kappa chain). To avoid inappropriate glycosylation when expressed in mammalian cells, asparagine-X-serine/threonine N-glycosylation sequons were mutated to asparagine-X-alanine [29]. Genes were then codon optimized for mammalian expression. For three of the very large proteins from the cysteine-rich modular protein family (CRMP1, 3 and 4), two to three constructs were designed, together spanning the ectodomain. Given the importance of CSP and its known domain architecture, both a full-length construct and N-terminal and C-terminal domain constructs were designed and used in this study. The remaining coding sequences were synthesized by Twist Biosciences or, for large or challenging genes, ThermoFisher.

Selection of proteins for inclusion in the human hepatocyte surface protein library proceeded similarly, using available proteomic, transcriptomic and functional data. The starting point for this was a manually-curated list of human cell-surface proteins. Cross-referencing was performed this with three human primary hepatocyte or hepatoma cell line proteomic data-sets [30–32] to identify around 1000 cell surface proteins with reasonable proteomic evidence of hepatocyte expression, adding a further 150 proteins which were not detected by mass spectrometry but were abundant in hepatocyte transcriptomes [33]. Because AVEXIS depends upon expression of ectodomains as soluble proteins, the majority of multi-pass transmembrane proteins were excluded from the list, retaining type I, II and III and GPI-anchored single-pass transmembrane proteins, plus a small number of multi-pass proteins with substantial N-terminal ectodomains (typically > 100 amino acids). Proteins with extremely large ectodomain coding sequences (> 5 kb) were also discarded, due to anticipated difficulty of gene synthesis. The resulting list of proteins was manually reviewed and the 189 proteins with the strongest evidence of abundant expression on the hepatocyte surface were selected for study (Additional file 1: Fig. S2). Plasmids encoding 127 of these (though with different tags from our prey configuration) were already available in previously designed libraries [15, 34, 35]. As exceptions to general rule of excluding multi-pass transmembrane proteins, extracellular domains of CD81 and SR-BI were included, as proteins known to have roles in sporozoite-hepatocyte invasion [7, 8, 36]. For CD81, the larger of the protein’s two extracellular loops was used. A similar construct has previously been shown to retain the ability to bind hepatitis C virus E2 [37]. For SR-BI, the entire extracellular loop was used.

For the 62 selected human proteins for which plasmids with coding sequences were not available, hepatocyte protein ectodomain coding sequences were designed as for the sporozoite library, with the exceptions that amino acid sequences were obtained from Uniprot, and that endogenous signal peptides and N-glycosylation sequons were retained. Mouse immunoglobulin κ light chain signal peptides were added to the constructs encoding CD81, SR-BI and type III transmembrane proteins (which lack signal peptides). The library also included constructs encoding a number of integrin heterodimers, as previously described [5].

### Cloning and protein expression

**Fig.1:**
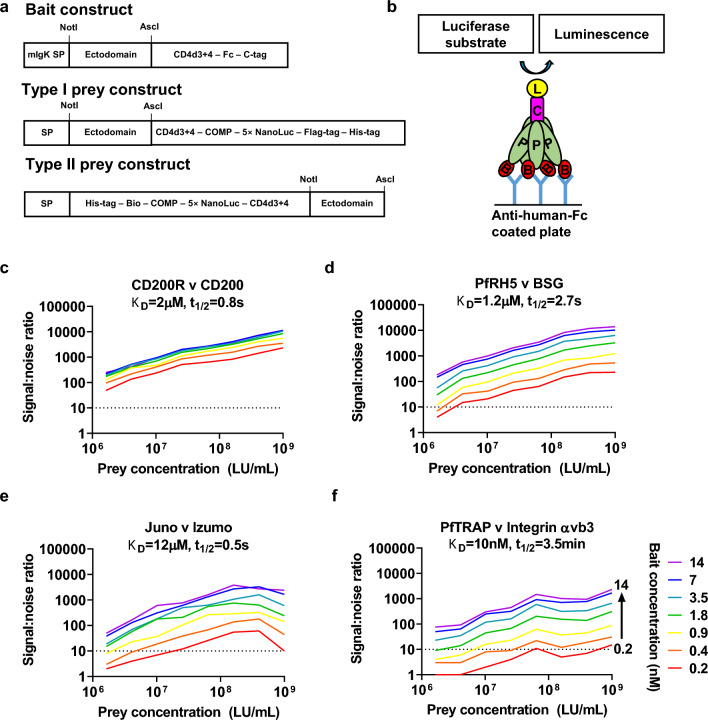
Development and validation of high-throughput modified AVEXIS. **a** and **b** Bait and prey expression constructs, and assay schematic. Baits contained a human Fc tag in place of the previously used biotin acceptor peptide. Preys contained a 5 × NanoLuciferase tag in place of the previously used beta-lactamase tag. ‘Type I’ and ‘Type II’ preys were used to allow selection of protein orientation (see Methods). Following protein expression, mostly in 24-deep-well plates to enhance throughput, the assay was performed as shown in panel B, with an Fc-tagged bait (labelled ‘B’; red) immobilized on a 96-well plate pre-coated with anti-human IgG-Fc mAb (blue), and probed for interaction with a interacting with a prey (labelled ‘P’; green) protein tagged with pentameric 5 × NanoLuc (labelled ‘L’; yellow). The rat cartilaginous oligomeric matrix protein pentamerization domain (labelled ‘C’; pink) mediates pentamerization. **c–f** Performance characteristics of the AVEXIS assay were validated using four pairs of proteins with known interaction affinities and dissociation half-lives [5, 15, 49, 65] as indicated on panel labels (for each pair, the bait is named before the prey; for full details, see methods). Graphs depict signal:noise ratio (Y-axis) for each pair at a range of prey concentrations ranging from 1 × 10^9^ LU/mL to 1.6 × 10.^7^ LU/mL (indicated on X-axis) and a range of bait concentrations ranging from 14 nM to 0.2 nM (indicated by coloured lines, as labelled on **f**. Each data point on these graphs corresponds to a single test of the interaction between a bait and prey at a specific concentration; data shown is representative of two similar experiments on separate days producing similar results. Signal:noise ratios were calculated as described in Methods, by reference to results with the same prey protein/concentration and an irrelevant bait (CD200R, except for CD200 prey, for which Juno was used as irrelevant bait). The dashed horizontal line represents signal:noise ratio of 10

Sporozoite ectodomain coding sequences were cloned using NotI/AscI restriction enzymes into a pTT5-based vector [38], in frame with sequence encoding tags as shown in Fig. 1A. Consequently, human Fc tagged bait proteins are expressed as dimers. Hepatocyte ectodomain coding sequences were cloned similarly into the prey vectors by using NotI/AscI restriction enzymes. Two prey vectors were used, according to the expected orientation of the native protein relative to the cell membrane. The majority of constructs, encoding proteins with free N-termini and with transmembrane domains C-terminal to the ectodomain, were cloned into a ‘Type I’ vector, providing tags as shown in Fig. 1A. Type II transmembrane proteins, with free C-termini, were expressed from a ‘Type II’ vector, again as shown in Fig. 1A. In the case of integrins, α chain ectodomains were cloned into the Type I prey vector, while β chain ectodomains were expressed without tags.

For recombinant protein expression, ectodomain constructs were transiently transfected using Expifectamine into Expi293F suspension cells as per the manufacturer's instructions (ThermoFisher). Transient transfections were performed in deep 24 well plates (Axygen), with 4 mL cells/well and transfected cells were maintained at 37 °C, 700 RPM, 8% CO2 and 70% relative humidity. Integrin preys were produced by co-transfection with α and β chain constructs. Supernatants were collected on day 3–4 post-transfection and secreted proteins in the supernatants quantified. Selected baits and preys which were expressed in 4 mL cultures at insufficient levels for AVEXIS were expressed in 25 mL cultures in Erlenmeyer flasks, and concentrated by using 30 kDa molecular weight cut-off (MWCO) centrifugal filters (Vivaspin, GE Healthcare).

ΔPf34 was designed as a fragment of Pf34, comprising a domain (residues 140–248) which is conserved among *Plasmodium* species [39]. PfRON6 N-terminal (residues 16–226) and C-terminal fragments (537–950) were designed by eliminating low-complexity regions of PfRON6. ΔFGFR4 was produced by deleting the Ig1 domain (as previously described [40]) from FGFR4 prey vector. Construct boundaries and sequences are listed in Additional file 2: Table S1 and Additional file 3: Table S2.

To produce purified monomeric protein for surface plasmon resonance (SPR), a codon-optimized FGFR4 ectodomain coding sequence with C-terminal CD4d3 + 4-His_6_ tag sequence was cloned into pTT5 [41]. Transfection of Expi293F cells was performed as above. Purification was performed using an AKTA Purifier instrument (GE Healthcare) and HiTRAP Talon column (GE Healthcare). Quality of all purified proteins was confirmed by means of Coomassie staining of an SDS-PAGE gel, demonstrating the expected molecular weight. The full-length Pf34 was determined to have a purity of 88%, with other proteins present believed to be breakdown products of Pf34 and the purity of FGFR4 is > 95% as assessed by densitometric analysis of Coomassie stained gels.

To produce LD1 monoclonal antibody (mAb), LD1 VH and VL coding sequences were synthesized [42] (Twist Bioscience) and cloned into mammalian expression plasmids (pVIPENTR, a kind gift of Martino Bardelli). Soluble LD1 mAb was expressed by transiently co-transfecting LD1 heavy and light chain constructs into Expi293F cells. Secreted LD1 mAb was purified by using Protein G columns (Pierce) on AKTA purifier (GE), and stored at − 80 °C.

### Bait and prey quantification and normalization

**Fig. 2:**
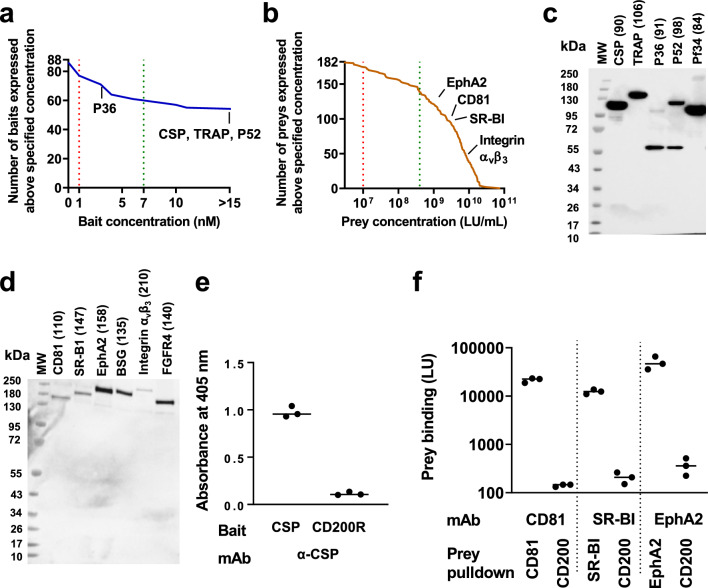
Expression of the *P. falciparum* sporozoite and human hepatocyte surface protein libraries. Panels **a** and **b** depict expression levels of bait (by ELISA) and prey (by luciferase assay) respectively, summarized as inverse cumulative distribution functions, and with concentrations of selected proteins of particular interest indicated by name. In the case of 54 relatively weakly-expressed baits and the integrin preys, for which transfections were performed in flasks, the results shown are those obtained after concentration of supernatant. Vertical dashed lines indicate boundaries between optimal, informative and less informative concentrations, as defined based upon the assay validation experiments (see Results and Fig. 1C–F). Panels **c** and **d** show Western blots of selected constructs: CSP, TRAP, P36, P52, Pf34 baits (detected with anti- C-tag antibody) and CD81, SR-BI, EphA2, integrin α_v_β_3_ and FGFR4 preys (detected with anti FLAG-tag antibody; the integrin β-chain is untagged and so not seen). Legend indicates expected molecular weight of each construct in kDa, including tags but excluding post-translational modifications. Some proteins were detected at higher molecular weights than expected, probably due to glycosylation. For Western blots of other proteins, see Additional file 1: Figs. S1 and S2, and for complete list of baits and preys with details of concentrations and Western blot results, see Additional file 2: Table S1 and Additional file 3: Table S2. The band seen at c. 55 kDa in blots of pre-concentrated baits is believed to represent reactivity of the anti-Ctag antibody with a HEK293-cell protein (rather than degraded bait) as it was also seen in supernatant from cells transfected with irrelevant constructs. **e** and **f** graphs depict the quantified levels of captured bait and preys using ELISA and luciferase assay, respectively

Fc-tagged baits were expressed as soluble proteins and quantified by ELISA. For ELISA, Nunc Maxisorp 96 well plates were coated with 50 ng/well of a high affinity mouse anti-human Fc monoclonal antibody (mAb clone R10Z8E9, Abingdon Health) in PBS and incubated overnight at 4 °C. Plates were washed 5 times in PBS containing 0.05% Tween 20 (PBS/T) and once in PBS. Plates were blocked with 5% skimmed milk in PBS/T for 1 h at room temperature. Blocking solution was removed and baits were immobilized onto the plate by incubating for 2 h at room temperature. After washing again, 50 µL/well of alkaline phosphatase-conjugated donkey anti-human Fc antibody (Jackson ImmunoResearch, Cat. No. 709-055-098) diluted 1:1000 in PBS was added and incubated for 1 h at room temperature. After further washing, 100 µL/well of freshly prepared P-nitrophenyl phosphate substrate (Sigma) in diethanolamine buffer was added and incubated for 10–15 min at room temperature. OD 405 nm was read by a Clariostar plate reader (BMG Labtech) and concentrations of unknown proteins were determined by interpolating from a standard curve of samples of known protein concentration (Fig. 2A). For use in AVEXIS screening, bait concentration was adjusted to a target of 7 nM by dilution with Blocker Casein (ThermoFisher) or concentration using a 30 kDa MWCO Vivaspin centrifugal filter.

Preys were expressed as soluble 5 × NanoLuc tagged proteins. Prey levels were quantified in supernatants using the Nano-Glo Luciferase Assay System according to the manufacturer’s instructions (Promega), with the exception that the substrate solution was diluted 1 in 20 in PBS prior to use. 50 µL of supernatant diluted 1:10,000 with casein was mixed with 50 µL of NanoLuc substrate in a well of 96-well white Maxisorp plate (VWR) and incubated for 3 min at room temperature. Plates were transferred to the Clariostar plate reader and luminescence units (LU) measured (Fig. 2B).

Subsequently, all preys were diluted with one volume of Blocker Casein, a step found to effectively reduce background noise (data not shown) or, in the case of proteins at ≥ 8 × 10^8^ LU/mL, adjusted to a target of 4 × 10^8^ LU/mL by dilution with Blocker Casein. Given that integrin constructs were of particular interest and these preys expressed at relatively low levels, integrin-containing supernatants were concentrated to ≥ 8 × 10^8^ LU/mL using a 30 kDa MWCO Vivaspin centrifugal filter, prior to addition of casein; other weakly expressed proteins were not pre-concentrated.

### Western blotting

Proteins were mixed with NuPAGE reducing sample buffer and boiled at 70 °C for 10 min and separated by SDS-PAGE on NuPAGE 4–12% Bis–Tris gels in anti-oxidant buffer (all from ThermoFisher). Resolved proteins were transferred to a nitrocellulose membrane using the Trans-blot Turbo system (BioRad). Membranes were probed with either biotinylated anti-C-tag conjugate (ThermoFisher, Cat. No. 7103252100) for the detection of baits, or biotinylated anti FLAG antibody (Sigma Aldrich, Cat. No. F9291) for detection of preys. Streptavidin-HRP (Pierce, Cat. No. 21130) was used as a secondary reagent. Proteins on the probed membrane were detected using SuperSignal West Pico Chemiluminescent substrate (ThermoFisher) (Fig. 2C, D).

### Antibody reactivity of selected baits and preys

Full length CSP bait was captured from expression supernatant onto a 96 well plate coated with anti-CSP mAb 2A10 (MR4 Resources), with supernatant containing CD200R bait used as a negative control. Captured CSP bait was detected by using ELISA as explained above.

CD81, SR-BI and EphA2 preys were captured from expression supernatant onto a white 96 well plate coated with cognate mAbs (1D6 [Abcam] for CD81, EP1556Y [Abcam] for SR-BI, MAB3035 [R&D Systems] for EphA2), with supernatant containing CD200 prey used as a negative control. Immobilized preys were quantified using luciferase assay as explained above.

### Protein interaction assays by AVEXIS

White Nunc Maxisorp 96 well plates (VWR) were coated with 50 ng/well mouse anti-human Fc monoclonal R10Z8E9 in PBS and incubated overnight at 4 °C. Plates were washed 5 times in PBS containing 0.05% Tween 20 (PBS/T) and once in PBS. Plates were blocked with 5% skimmed milk in PBS/T for 1 h at room temperature. Blocking solution was removed and normalized baits were immobilized onto the plates overnight at 4 °C. The next day, plates were washed as above, and normalized preys were added and incubated for 2 h at room temperature. Plates were washed and bound preys were detected by using the Nano-Glo Luciferase Assay System (as described for prey quantification).

Given that background levels of signal were observed to vary between preys, the screen was performed by testing one prey against all baits on a single plate. Each plate included the following controls: a ‘pulldown’ of the prey being investigated using anti-FLAG antibody (Sigma Aldrich, Cat. No. F1804), to confirm the addition of functional prey; positive control interaction using *P. falciparum* RH5 as bait and human BSG as prey; and a negative control interaction using irrelevant bait and human BSG as prey.

AVEXIS results are presented in terms of signal:noise ratios, to correct for varying levels of non-specific luminescence attributable to different constructs. The major determinant of the level of noise is the prey (background luminescence after application to wells coated with irrelevant baits varies tenfold or more between different preys). In the case of experiments using small numbers of baits and preys (< 10 in total), signal:noise ratios were thus calculated simply by dividing by the result obtained in wells containing an irrelevant bait and probed with the prey of interest. In the case of the high-throughput screen, a ‘single correction’ was performed initially, dividing luminescence by the median result across all baits probed with the prey in question (i.e. the median result on the plate). The use of the median as ‘noise’ is based upon the assumption that the vast majority of protein pairs will *not* interact, and hence the median bait can be regarded as a more representative irrelevant control than any single bait picked to act as a control. A small number of baits appeared to bind non-specifically to multiple preys. Therefore, ‘double correction’ was performed, dividing the ‘single corrected’ result for a given interaction by the median ‘single corrected’ result for all preys tested with the same bait to produce a final signal:noise ratio.

The screen was performed once. All apparent novel interactions with signal:noise ratios exceeding 30 were repeated, as described in Additional file 5: Table S4, initially using the same protein preparations. Interactions which were not reproduced were assumed to have been falsely positive in the initial screen, probably due to incomplete plate washing.

Interaction blocking experiments were performed similarly, with the exception that blocking reagents (antibodies) were added onto the immobilized baits on the plate for 1 h prior to addition of preys.

### Surface plasmon resonance

Surface plasmon resonance studies were performed at an analysis temperature of 25 °C using a Biacore T200 instrument and HBS-EP + buffer (both from GE Healthcare). Mouse anti-human Fc monoclonal antibody R10Z8E9 was immobilized onto active and reference flow cells of a Series S sensor CM5 chip using an amine capture kit (both from GE Healthcare). Approximately 1000 response units of Pf34 bait protein was captured onto the active flow cell, with a molar equivalent quantity of PfRH5 bait as a control protein bearing the same tags (i.e. CD4d3 + 4, human Fc and C-tag).

For use as analyte in the mobile phase, FGFR4 protein bearing CD4d3 + 4, biotin acceptor peptide and His_6_ tags was purified by affinity chromatography as above. Analytical size exclusion chromatography using a Superdex 200 Increase 10/300 column confirmed the absence of aggregates from the protein preparation (GE Healthcare).

Increasing concentrations of the purified FGFR4 analyte were injected at 30 µL/min over the chip surface, each for 60 s followed by a 120 s dissociation phase. The Biacore single-cycle kinetics mode (without regeneration between injections) was used, although due to the rapid kinetics of the interaction, all previously bound protein was dissociated prior to the start of each injection.

To determine Pf34-PfRON6 kinetics and affinity, PfRON6 C-terminal bait was captured onto the active flow cell, and purified monomeric Pf34 protein bearing CD4d3 + 4, biotin acceptor peptide and His_6_ tags was used as analyte. Increasing concentrations of Pf34 analyte were injected at 30 µL/min over the chip surface, each for 300 s followed by a 600 s dissociation phase. The Biacore multi-cycle kinetics mode was used, with regeneration with 3 M MgCl_2_ between injections.

Data was analysed using Biacore T200 evaluation software (GE Healthcare). All data was double-reference subtracted before model fitting (i.e. both the signal on the reference flow cell with the same analyte and the signal detected during a buffer-only blank injection were subtracted from the signal with the analyte on the active flow cell). 1:1 binding models were fitted to both kinetic data and equilibrium binding data.

To test whether heparin and heparan sulfate (Sigma) affected the Pf34-FGFR4 interaction, each was mixed with FGFR4 analyte to a final concentration of 1 mg/mL in HBS-EP + buffer, then injected over Pf34 ligand that was captured onto Fc coated chip surface.

### *Plasmodium falciparum* in vitro inhibition of sporozoite invasion (ISI) and blood-stage growth inhibitory activity (GIA) assays

Salivary glands from *P. falciparum*-infected female *Anopheles stephensi* mosquitoes were dissected in William’s B medium [43] and disrupted using a plastic pestle. Primary human hepatocytes isolated from surgical liver segments [43] were seeded in 96 well tissue culture plates (Corning) at a density of 62500 per well and cultured at 37 °C, 5% CO_2_ in William’s B medium for 48 h before inoculation of sporozoites.

To investigate the effect of anti-FGFR4 antibodies, primary hepatocytes were incubated with the appropriate reagent (as described in legend for Fig. 5 and Additional file 1: Fig. S5) for 30 min before addition of sporozoites. To investigate the effect of anti-Pf34 antibodies, sporozoites (at a 1:1 MOI) were pre-incubated with the appropriate reagent (as described in legend for Fig. 5 and Additional file 1: Fig. S5), on ice, for 30 min before addition to hepatocytes. Purified IgG from naïve rabbit serum was used as the control in this study, and the percentage of inhibition is relative to the control group.

Following sporozoite inoculation, hepatocytes were incubated at 37 °C, 5% CO2. Media was replaced at 3 h after sporozoite incubation and then daily. NF54 and NF175 infected hepatocytes are fixed 3- or 5-days post infection respectively with 4% PFA and stained with either PfGAPDH (1:50.000, European malaria reagent repository, Cat. No. 7.2) or PfHSP70 (1:75, StressMarq Biosciences, Cat. No. SPC-186) or both to visualize the exo-erythrocytic forms (EEFs) as described earlier [43]. Number of EEFs were quantified in duplicate or triplicate wells using Leica DMI600B high content microscope at 20 × objectives: a tile size of 9 × 9 was obtained per well.

Assays of blood-stage GIA were performed, as previously reported [44] using anti-Pf34 rabbit pAbs using the concentrations shown in (Additional file 1: Fig S5C) against 3D7 clone *P. falciparum.*

### Generation of Pf34 and PfRON6 conditional knockout parasites

Culturing, maintenance and transfections *of P. falciparum* asexual blood stages were performed as describe earlier [45]. *Plasmodium falciparum* conditional knockout (cKO) parasites were generated by transfecting NF54-DiCre parasites with 35 µg of repair plasmid, and 20 µg of one or two pDC2-Cas9-U6-hdhfr vectors (as specified below), each encoding the Cas9 enzyme and a gene-targeting single guide RNA (sgRNA). Repair plasmids were synthesized in pMX vector (ThermoFisher).

For Pf34 cKO parasites, the repair construct consisted of (1) 5’ homology region (HR) spanning − 242 bp to + 281 bp of coding sequence, followed by a 103 bp loxP containing SERA2 intron (loxPint) cassette, (2) recodonized Pf34 CDS from + 43 bp to the stop codon, followed by loxP element, (3) and 3’ HR (Additional file 1: Fig S6A). An XmaI restriction site was included after signal peptide to facilitate subsequent modification.

For Pf34-3XHA-GFP cKO parasites, a triple HA tag coding sequence was inserted after the Pf34 signal peptide in the above construct (using XmaI restriction enzyme), and eGFP coding sequence was inserted after the silent loxP cassette using AvrII and SacII restriction enzymes (Additional file 1: Fig S6B).

For a second attempt at production of Pf34 cKO (but not Pf34-3XHA-GFP cKO) parasites, a further repair construct, designated ‘scarless’ was produced: this contained recodonized sequence encoding the wildtype Pf34, without the XmaI site.

For PfRON6 cKO parasites, the repair construct consisted of (1) 5′ HR spanning − 245 bp to + 251 bp of coding sequence, followed by 103 bp loxPint cassette, (2) recodonized PfRON6 CDS from + 41 bp to till stop codon, followed by loxP element, (3) and 3’ HR (Additional file 1: Fig. S7A). The PfRON6-3XHA-GFP repair construct was generated by incorporating triple HA tag coding sequence immediately before the stop codon, and eGFP coding sequence after the silent loxP cassette, using AvrII and SacII restriction enzymes (Additional file 1: Fig. S7B).

sgRNA sequences were identified using Eukaryotic Pathogen CRISPR guide RNA/DNA Design Tool (EuPaGDT, http://grna.ctegd.uga.edu/) and cloned into pDC2-Cas9-U6-hdhfr as described earlier [46]. Briefly, a pair of phosphorylated complementary oligonucleotides including the desired guide sequence were annealed and ligated into BbsI digested pDC2-Cas9-U6-hdhfr vector. Oligonucleotide sequences are listed in Additional file 6: Table S5.

For the generation of Pf34 cKO parasites, multiple rounds of transfections were performed employing various constructs and sgRNAs in the following sequence: (1) Pf34 cKO construct + one sgRNA, (2) Pf34-3XHA-GFP construct + one sgRNA, and (3) Pf cKO scarless construct + two sgRNAs. For the generation of PfRON6 cKO parasites, PfRON6 cKO construct and PfRON6-3XHA-GFP with two sgRNA plasmids were used in each transfection.

Highly synchronized schizonts at 48 h post-invasion (hpi) were transfected using an Amaxa electroporator and Lonza 4D-Nucleofector kit with P3 primary cell buffer. Selection with 2.5 nM WR99210 drug was started 24 h after transfection and renewed daily for 4 days. Once infected RBCs were visible by Giemsa staining, site-specific recombination at both 5’ and 3’ ends was confirmed using two primer pairs, P1/P5 and P4/P6. After confirming the integration, limiting dilution was performed in 96 well plates with 0.3 parasites per well, followed by outgrowth for 12 days. A diagnostic PCR was performed on clonal populations, using primer pairs P1/P2 and P3/P4 to confirm the absence of non-transfected parasites. Primer sets used to confirm successful integration and absence of non-transfected parasites are listed in Additional file 6: Table S5 and indicated diagrammatically in Additional file 1: Figs. S6 and S7. To induce gene excision, these transgenic parasites were treated at ring stage with 100 nM rapamycin for 4 h while shaking. DMSO was used to generate unexcised control parasites. After treatment, both DMSO and RAP-treated parasites were washed 3 times with 10 ml RPMI-1640 and returned to culture.

### Parasite growth kinetics

To measure parasite growth kinetics, NF54-Dicre (wild type control parasites), PfRON6 cKO and PfRON6-3XHA-GFP cKO parasites were tightly synchronized as described earlier [47], and grown in culture media with either DMSO or 100 nM rapamycin. Cultures were grown in three 96 well plates, started with 0.5% parasitaemia. Every 24 h, 20 µL of sample was collected, spun down to remove culture medium and fixed in 2% paraformaldehyde/0.2% glutaraldehyde in PBS. Parasite nuclei were stained with Sybrgreen and parasitaemia was counted by flow cytometry on a LSRFortessa flow cytometer (Becton Dickinson). Flow cytometry data were analysed using FlowJo10 analysis software.

## Results

### AVEXIS assay development for systematic high-throughput screen

The AVEXIS assay has previously been used to identify protein–protein interactions, including those with low-affinity and fast dissociation rates [16]. The method has been subsequently modified to enhance sensitivity by employing luciferase instead of β-lactamase as the reporter assay[48]. Here, given the limited prior information about candidate sporozoite ligands and hepatocyte receptors and the large number of expressed candidate proteins, a decision was made to perform a broad screen and, therefore, further modified the method to enhance throughput while preserving or improving sensitivity (Fig. 1A, B). The assay modifications allowed expression of most proteins in 24-well plates, removed dependency on streptavidin–biotin-mediated capture and so eliminated the need for bait purification or dialysis and expensive streptavidin-coated plates, and retained the sensitive and immediate luciferase-generated luminescence readout. 

The modified assay was initially validated by testing its ability to detect four known protein–protein interactions. The modified assay remained capable of demonstrating these interactions (Fig. 1C, F), which included *P. falciparum* RH5: human basigin [15] and mouse Juno: Izumo (which, in monomeric format, is strikingly weak [*K*_D_ =  ~ 12 µM] and transient [t_1/2_ =  ~ 0.5 s] [49]).

All interactions were detected with a high signal:noise ratio when using bait concentration of 7 nM and prey concentration 4 × 10^8^ LU/mL, and all remained detectable with substantially lower protein concentrations, albeit with lower signal:noise ratios (Fig. 1C, F). There was not an obvious relationship between assay sensitivity and interaction affinity or half-life. Sensitivity may instead be influenced by protein quality (e.g. fraction of proteins with correct conformation) and the accessibility of binding sites within these particular constructs.

### Creation of sporozoite and hepatocyte surface protein ectodomain library

Available proteomic, transcriptomic and functional data were reviewed to assemble lists of 84 *P. falciparum* proteins and 189 human proteins which are likely to be expressed on the sporozoite and hepatocyte surfaces respectively, and had protein architectures amenable to AVEXIS (see Methods).

The sporozoite protein set included 45 proteins included in our previously-reported library [17]. 13 proteins included in that library were excluded from the current study (on the basis of weak evidence of sporozoite expression, or functional/homology evidence suggesting they were unlikely to be ligands for host receptors (e.g. a putative vacuolar protease, a redox protein, and members of the vesicular trafficking p24 family). 45 of the sporozoite constructs were newly designed for this study. The very large cysteine rich modular proteins CRMP1, 3 and 4 [50] were split into two to three fragments, and three different CSP-based constructs were designed, resulting in a total of 90 sporozoite constructs. Of the 189 human proteins, 127 constructs were taken from previously designed libraries [15, 34, 35] while the remaining 62 were newly designed for this study.

Out of these constructs, a total of 88 sporozoite constructs and 182 hepatocyte proteins were successfully cloned and attempts were made to express them as AVEXIS baits and preys respectively (i.e. generic construct design as shown in Fig. 1A). Synthesis of the remaining coding sequences was unsuccessful as they might be toxic inserts or having repetitive regions. Full details of constructs produced are provided in Additional file 2: Table S1 and Additional file 3: Table S2.

Bait expression levels obtained, as quantified by ELISA (Fig. 2A), and examples of the protein quality as assessed by Western blot are shown in Fig. 2C (see also Additional file 1: Fig. S1, and Additional file 2: Table S1). 82 baits were obtained at concentrations > 1 nM which were considered as potentially informative (based on the results obtained with the test set of known interactions [Fig. 1C–F]). 61 baits (including CSP, TRAP and P52) were obtained at > 7 nM, which were considered as optimal. To achieve these levels, spin filter concentration was required for 54 baits. As concentration measurements were based upon ELISA using antibodies to the C-terminal Fc-tag, they would not necessarily be accurate in cases in which there was appreciable protein degradation, as was seen for some baits (Additional file 1: Fig. S1). 

Of the preys, 165 were obtained at concentrations > 1 × 10^7^ LU/mL which were considered as potentially informative, of which 139 preys (including the CD81 large extracellular loop, SR-BI ectodomain, EphA2, and integrin α_v_β_3_) were obtained at > 4 × 10^8^ LU/mL (Fig. 2B) which were considered as optimal based upon our results with the test set of known interactions. Results of Western blotting of preys are shown in Fig. 2D and Additional file 1: Fig. S2 (see also Additional file 3: Table S2).

To provide additional assurance regarding the quality of key bait and prey proteins, particularly the activity of the folded proteins in a plate-format assay, the full-length CSP bait, and CD81, SR-BI, and EphA2 preys were further tested to determine whether they could be captured onto a 96-well plate using appropriate monoclonal antibodies. Captured baits and preys were detected using ELISA and luciferase assay respectively, demonstrating the expected antibody reactivity (Fig. 2E, F).

### AVEXIS identifies sporozoite ligand: hepatocyte receptor interactions

**Fig. 3:**
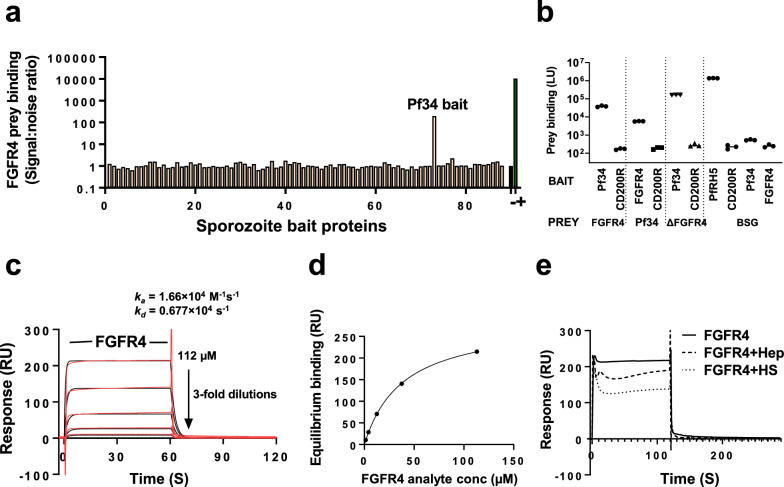
Human FGFR4 interacts specifically with Pf34. **a** Human hepatocyte FGFR4 receptor ectodomain prey was tested by AVEXIS for binding to a library of 88 *P. falciparum* ligand ectodomains. Bait numbers correspond to named proteins in Additional file 2: Table S1. ‘ + ’ and ‘−‘ indicate PfRH5 bait/BSG prey and CD200R bait/BSG prey, used as positive and negative controls, respectively. **b** The Pf34-FGFR4 interaction was confirmed by re-testing in three independent AVEXIS experiments, including testing with the reverse orientation of proteins from that used in the screen. An FGFR4 construct lacking the first immunoglobulin domain (ΔFGFR4) remained capable of interacting with Pf34, with slightly increased signal:noise ratio. Bars represent median ± range of the three experiments. **c** and **d** show binding of a threefold dilution series of FGFR4 analyte (in solution) to Pf34 ligand (immobilized on chip) by surface plasmon resonance. Raw data and results from a duplicate experiment with independent protein samples are shown in Additional file 1: Fig. S4A–E, H: the duplicate demonstrated overall similar binding with somewhat higher estimated affinity due to more rapid association. Panel **c** shows kinetics, with observed binding (red lines, double-reference-subtracted sensorgrams) overlaid with results of fitting a 1:1 interaction kinetic model (black lines). Panel **d** shows equilibrium binding levels (points, from the experiment shown in **c**) with the results of fitting a 1:1 equilibrium binding model (line). Curvature indicates binding tending towards saturation, consistent with a specific interaction. **e** shows no increase in Pf34-FGFR4 interaction when 1 mg/mL heparin (hep) or heparan sulfate (HS) were pre-mixed with 100 µM FGFR4 analyte before being injected for 120 s over immobilized Pf34 ligand. Graphs shown here are double-reference-substracted i.e. report the change in active minus reference flow cell response between injection of the FGFR4-containing analyte, and injection of control sample comprising buffer only, Hep only, or HS only. Based upon single-reference-subtracted sensorgrams (active minus reference flow cell, not shown), there was no binding of any of the non-FGFR4-containing control samples to Pf34

Having developed the modified high-throughput AVEXIS method and constructed the candidate sporozoite ligand and hepatocyte receptor libraries, a systematic screen for ligand-receptor interactions was performed (Fig. 3A and Additional file 4: Table S3). Signal:noise ratios were calculated by initially correcting for noise attributable to the prey, and then for noise attributable to the bait (see Methods). Raw, prey-corrected and final results are shown on separate worksheets in Additional file 4: Table S3.

All 16016 possible sporozoite protein/hepatocyte protein pairs were tested (Additional file 4: Table S3). Of these, 7540 candidate interactions were tested using protein concentrations which were considered as optimal (bait concentration ≥ 7 nM, prey concentration 4 × 10^8^ LU/mL, and good protein quality as assessed by Western blotting), and a further 4718 were tested using protein concentrations which our assay validation data (Fig. 1C–F) suggested would provide a signal:noise ratio of > 10 for any of our ‘test set’ of four known interactions.

Across the tested interactions, the highest signal:noise ratio (198) was observed with the combination of the sporozoite protein Pf34 and the human cell-surface protein FGFR4 (Fig. 3A). A novel and reproducible interaction was also observed between PIESP15 and LDLR. The protein PIESP15 was under investigation in a separate study by an overlapping team of authors [17] and so this interaction was not explored further here. Full results of the screen, and a summary table of additional protein pairs with a signal:noise ratio exceeding 5, are presented in Additional file 4: Table S3 and Additional file 5: Table S4. No detectable interactions were observed with any of the proteins which have been most prominently implicated as possible sporozoite ligands (CSP, TRAP, P36, P52) or hepatocyte receptors (CD81, SR-BI, EphA2), apart from the known interaction of TRAP with α_v_ integrins [5].

### Interaction of Pf34 with FGFR4 is specific and consistent with 1:1 binding kinetics

Pf34 (PF3D7_0419700) is a GPI-anchored protein expressed by all parasite stages and localized to rhoptry necks in blood-stage parasites [39]. Pf34 has orthologs in rodent parasites, but to date, there are no studies demonstrating the role of this protein in sporozoite invasion of hepatocytes. Its *Plasmodium berghei* ortholog (PBANKA_0721800) was not covered in recent large-scale ‘PlasmoGEM’ screens of gene essentiality in the *P. berghei* blood, mosquito, and pre-erythrocytic stages [51, 52].

FGFR4 (CD334) is most strongly expressed in the liver, where it is the dominant FGFR family member [30]. The full-length FGFR4 splicing isoform has an extracellular domain which consists of 3 immunoglobulin-like domains, a single transmembrane domain and a cytoplasmic tyrosine kinase domain [53]. A liver-specific signalling pathway through FGFR4, stimulated by ligands including FGF19, is involved in regulation of cholesterol and bile acid metabolism [54]. FGFR4 has previously been implicated in liver stage development of *Plasmodium yoelii* in Hepa1-6 cells, as one among a number of ‘hits’ in a screen investigating the role of host kinases in EEF development [55], although negative results were obtained in a similar screen investigating *P. berghei* [56].

To confirm the Pf34-FGFR4 interaction, the AVEXIS assay was repeated using reciprocally-oriented constructs, with FGFR4 expressed as dimeric bait and probed with pentameric Pf34 prey. Again, clear and reproducible binding was observed (Fig. 3B).

To demonstrate Pf34 and FGFR4 interact with the saturable Langmuir kinetics typical of a specific 1:1 interaction, surface plasmon resonance (SPR) was performed. Weak but clear saturable binding between Pf34 ligand and FGFR4 analyte was observed with an equilibrium binding constant (*K*_D_) of ~ 40 µM and rapid kinetics including t_1/2_ < 1 s (Fig. 3C, D, and Additional file 1: Fig S4A–E and H). Kinetic values approached the limits for determination using a Biacore T200 instrument and software, and inspection of residual plots suggested that fitted values may have underestimated association and dissociation rates. Re-fitting of the data (using GraphPad Prism) provided a good fit with similar *K*_D_ (46 μM) and t_1/2_ = 0.4 s. The ability of the AVEXIS assay to detect this extremely weak interaction further illustrates the power of the technique.

The extracellular region of FGFR4 protein contains three immunoglobulin-like (Ig-like) domains [57]. Previous studies show that the first of these, Ig1, plays a regulatory role and is not required for binding of endogenous human ligands such as FGF9 and FGF19 [40]. To test the same here in this study, a truncated form of FGFR4 lacking Ig1 (ΔFGFR4, Additional file 2: Table S2) was produced and demonstrated that, as for the endogenous ligands, Ig2 and Ig3 were sufficient for interaction with Pf34 (Fig. 3B).

The binding of many FGF family members to their receptors is known to be enhanced by heparin and/or heparan sulfate [58]. SPR was used to investigate whether heparin and heparan sulphate (HS) may have a similar affect upon the Pf34—FGFR4 interaction. A similar design to that used in the previous experiment measuring Pf34—FGFR4 kinetics was used, assessing whether pre-incubation of soluble monomeric FGFR4 with heparin or HS had any effect upon binding to Pf34 immobilized on the chip. No enhancement of FGFR4 binding to Pf34 was seen (Fig. 3E).

Having identified the interaction between human FGFR4 and *P. falciparum* Pf34, an investigation was undertaken to determine whether this interaction is conserved across species by testing murine FGFR4 for interaction with the Pf34 orthologs found in the rodent malaria parasites *P. yoelii* and *P. berghei.* No evidence of interaction was observed for either of these protein pairs, despite expression of all proteins at levels in the range expected to give optimal AVEXIS sensitivity (Additional file 1: Fig. S3A).

### Pf34 interacts with the C terminus of PfRON6 in AVEXIS

**Fig. 4:**
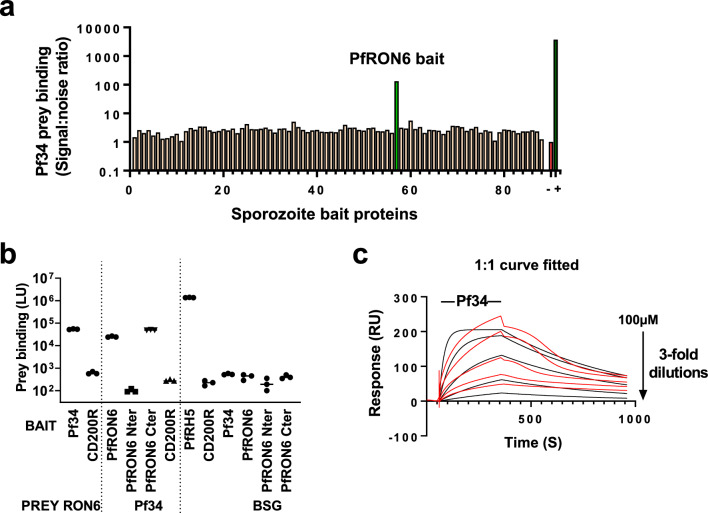
Exploration of additional Pf34-related interactions. **a** AVEXIS screening of Pf34 prey against the sporozoite ectodomain bait library demonstrates an interaction of Pf34 with PfRON6. **b** Further assessment of the Pf34-PfRON6 interaction by AVEXIS. The Pf34-PfRON6 interaction was detectable in both “bait-prey” orientations. PfRON6 N-terminal and C-terminal fragments were tested for their interaction with Pf34. The PfRON6 N-terminus did not interact with Pf34, while the PfRON6 C-terminal region, which containing conserved sequences, was sufficient for interaction with Pf34. Results shown here are from three independent experiments and bars represent median ± range of the three experiments. **c** Kinetics of interaction of soluble Pf34 analyte with immobilized PfRON6 C-terminal bait ligand in SPR. Observed binding (red lines, double-reference subtracted sensorgrams) is shown along with results of fitting a 1:1 interaction kinetic model (black lines). Increasing concentrations of Pf34 analyte by threefold dilution series were injected for 300 s over PfRON6 C-terminal ligand immobilized onto Fc sensor chip. Binding increased with increasing Pf34 analyte concentration, but saturable binding was not achieved even with 100uM Pf34. The fit of the 1:1 interaction model was poor. Raw data and further details are shown in Additional file 1: Fig S4F–H

Other sporozoite proteins which might interact with Pf34 were sought, as pairwise host–pathogen protein–protein interactions are frequently components of larger multi-molecular complexes (for example the PfRH5-RIPR-CyRPA complex, each member of which is essential for *P. falciparum* blood-stage invasion [59]). Pf34 protein was expressed as a pentameric luciferase-tagged prey and used AVEXIS to screen this prey for interaction with the full set of sporozoite protein baits. A weak but specific and reproducible interaction of Pf34 with PfRON6 (Fig. 4A) was identified. This interaction was also detectable in the reciprocal orientation (with Pf34 bait and PfRON6 prey; Fig. 4B). A number of strands of evidence suggest that PfRON6 may play an important role in *P. falciparum* host cell invasion: the PfRON6 gene is refractory to deletion in the asexual blood-stage, contains a sequence which is highly conserved across *Plasmodium* spp (ELKLKFEAMSRIKEYK) and a cysteine-rich domain which is conserved across *Apicomplexa*, and the protein is known to co-localize with Pf34 in the rhoptry neck (by confocal immunofluorescence microscopy) [60]. While RONs have been targeted in the sporozoite stage of *P. berghei *[61], RON6 has not been targeted, and no RONs have been targeted in *P. falciparum* [51, 52] till date.

PfRON6 has a large central low-complexity region. To determine which regions of PfRON6 are involved in the interaction with Pf34, we expressed PfRON6 N-terminal and C-terminal regions (Additional file 2: Table S1), excluding the low-complexity region. By AVEXIS, the C-terminal fragment was found to be sufficient for interaction with Pf34 (Fig. 4B). This fragment includes the conserved sequences described above*.* The N-terminal fragment did not interact with Pf34.

By contrast, no interaction was observed between a conserved fragment of Pf34 (ΔPf34, Additional file 2: Table S1) with full length PfRON6 or either of the above PfRON6 fragments (data not shown).

SPR was performed to determine kinetics and affinities of Pf34 and PfRON6 interaction, using the PfRON6 C-terminal fragment as the chip-captured ligand and Pf34 as the soluble analyte. A dose-dependent increase in binding was observed, but was not well-fitted by a 1:1 interaction model (Fig. 4C, Additional file 1: Fig. S4F–H). Possible explanations for this include heterogeneity of the PfRON6 ligand (Additional file 1: Fig. S3C), or an element of non-specific binding, although the AVEXIS data suggested a high degree of specificity of the interaction.

### Effect of anti-Pf34 and anti-FGFR4 antibodies upon protein interaction and sporozoite invasion

**Fig. 5:**
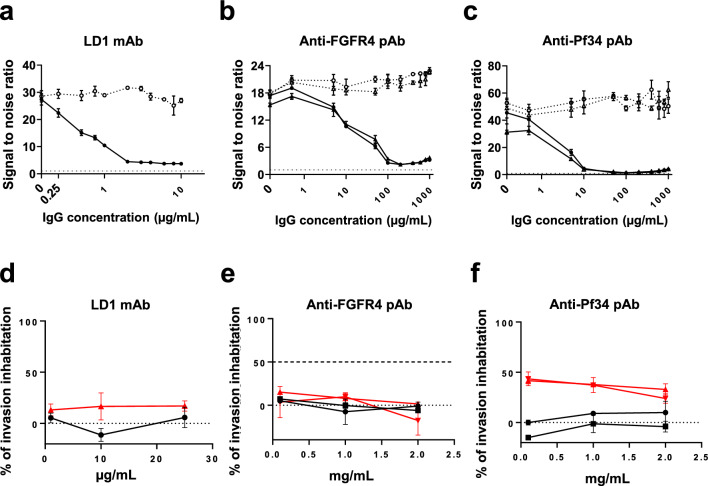
Dose-dependent inhibition of Pf34-FGFR4 interaction using anti-FGFR4 and anti-Pf34 antibodies, and effect upon sporozoite invasion. Panels **a** and **b** indicate the effect upon binding of Pf34 prey of pre-incubation of plate-bound FGFR4 bait with anti-FGFR4 antibodies at a range of concentrations (indicated by X-axis). Panel **a** shows results with LD1 mAb, panel **b** with anti-FGFR4 rabbit polyclonal IgG. Open symbols and dotted lines represent control sera, while closed symbols and solid lines represent mAbs or polyclonal immune IgG. For polyclonal samples, separate lines represent IgG from independent rabbit serum samples. Points represent median and error bars represent range of three technical replicate wells under each condition. Results are presented in terms of signal:noise ratio, with noise defined as LU of prey binding to irrelevant bait and hence a result of 1 indicating no binding. Similarly, panel **c** indicates the effect upon binding of FGFR4 prey of pre-incubation of plate-bound Pf34 bait with anti-Pf34 rabbit pAbs. Symbols, lines, points and error bars are as for **a** and **b**. Panels **d–f** show the effect of anti-FGFR4 and anti-Pf34 antibodies in an ISI assay using the NF175 parasite line. Panels D and E are each representative of duplicate experiments. The experiment shown in panel F was performed in singlicate. Black and red solid lines indicate sporozoite invasion inhibition using control antibodies and active antibodies respectively. The lower dotted line at 0% inhibition represents the mean level of invasion across all negative control antibodies used in the experiment. The upper dashed line indicates 55% inhibition observed using CD81 mAb at 25 µg/mL (positive control). Error bars indicate the median ± range from triplicate wells

To examine the role of Pf34-FGFR4 interaction in the invasion of hepatocytes by *P. falciparum* sporozoite, the effect of antibodies against Pf34 and FGFR4 was tested upon the protein interaction, and upon invasion of *P. falciparum* sporozoites into human hepatocytes. The invasion assay was considered successful when 1–4% of hepatocytes had EEFs in the control group.LD1 is a monoclonal antibody that inhibits interaction of FGFR4 with its natural ligands [62]. LD1 completely blocked the Pf34-FGFR4 interaction in AVEXIS at a concentration of 2 µg/mL (Fig. 5A), suggesting Pf34 may interact with FGFR4 near the binding site for the endogenous ligands.

Antibodies against Pf34 and FGFR4 were also raised in rabbits. By AVEXIS, dose-dependent inhibition of the Pf34-FGFR4 interaction was observed by these polyclonal antibodies (pAbs), with complete blockade at 100 µg/mL for total IgG from FGFR4-immunized rabbits, and 50 µg/mL for total IgG from Pf34-immunized rabbits. (Fig. 5B, C).

To evaluate the effect of blockade of the Pf34—FGFR4 interaction upon sporozoite—hepatocyte invasion, an inhibition of sporozoite invasion (ISI) assay was performed using NF54 and NF175 sporozoites, and human primary hepatocytes.

There was a little evidence of a dose-dependent effect of any of the three antibodies on NF175 parasites (Fig. 5D–F), although a non-dose-dependent inhibition of invasion was observed by the anti-Pf34 antibody from two independent rabbits in a single experiment. Some dose-dependent inhibition of NF54 sporozoite invasion by LD1 and anti-FGFR4 antibodies was observed, although this reached only 20% at the highest antibody concentrations tested (25 µg/mL LD1 mAb and 2 mg/mL anti-FGFR4 rabbit pAbs) (Additional file 1: Fig. S5A, B).

A minor effect of anti-Pf34 antibodies was observed upon blood-stage parasites in an assay of growth inhibitory activity (GIA) (Additional file 1: Fig. S5C).

### Conditional genetic disruption of Pf34 and PfRON6

A previous attempt to disrupt PfRON6 using conventional gene targeting approaches was unsuccessful, suggesting an indispensable role of PfRON6 in *P. falciparum* blood stages, and disruption of Pf34 appeared to have a substantial blood-stage fitness costs in a genetic screen [60, 63].

To attempt characterization of the roles of Pf34 and PfRON6 across all *Plasmodium* life cycle stages, a conditional gene disruption approach based on expression of the DiCre recombinase system was used, in which gene disruption is induced by rapamycin, in NF54 parasites [45, 46]. For each locus, attempts were made to produce two transgenic parasite lines: firstly ‘simple’ conditional knockouts, and secondly parasites in which the targeted protein would bear a 3xHA tag prior to DiCre-mediated recombination, and GFP would be expressed after recombination (Additional file 1: Figs. S6–S7).

For each of the two PfRON6 disruption strategies, a single round of transfection, WR99210 drug selection, and limiting dilution cloning resulted in two clones for which integration of the targeting vector and the absence of wild-type parasites was confirmed by PCR (Additional file 1: Figs. S6–S7).

No parasites were recovered after WR99210 selection for either of the two Pf34 disruption strategies, despite attempting two different repair plasmid and guide RNA design strategies for each.

### PfRON6 is essential for efficient blood-stage development

**Fig. 6:**
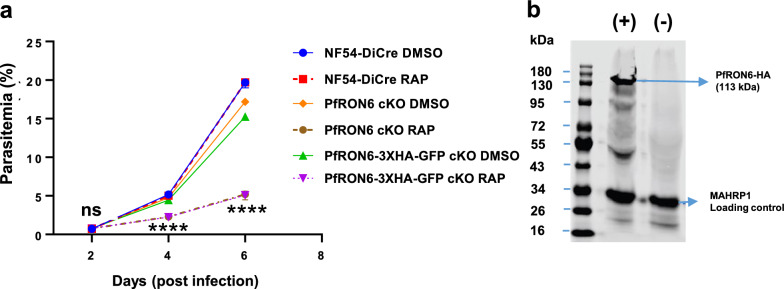
Asexual blood-stage growth kinetics of PfRON6 cKO and PfRON6-3XHA-GFP cKO parasites. **a** Asexual blood-stage growth curve over 6 days (3 replication cycles) of Nf54-DiCre, PfRON6 cKO, and PfRON6-3XHA-GFP parasites in culture medium with either DMSO or rapamycin (RAP). Error bars represent range of three technical replicate wells under each condition. Statistical significance was determined using a two-way ANOVA comparing PfRON6 cKO RAP and PfRON6-3xHA-GFP RAP to DMSO treated lines followed by Dunnett’s multiple comparison post-test. **** P < 0.0001. ns, not significant. **b** Western blot of PfRON6-3XHA-GFP blood-stage parasite material (with (+) or without (−) rapamycin treatment) was probed with anti-HA (3F10) antibody

Blood-stage growth kinetics studies were performed using PfRON6 cKO and PfRON6-3XHA-cKO parasites. These parasites grew at similar rates with the wild type NF54-DiCre in the absence of rapamycin (DMSO control). In concordance with the previously reported unsuccessful attempts to disrupt PfRON6 [60], a significant decrease in growth rate was observed when PfRON6 cKO or PfRON6-3XHA-GFP cKO parasites were treated with 100 nM rapamycin at early ring stage, whereas no effect was observed in NF54-DiCre-rapamycin treated parasites. A parasite reduction of > 75% in PfRON6-disrupted parasite cultures was observed after 3 erythrocytic growth cycles (144 h) when compared to non-PfRON6-disrupted NF54-DiCre control culture (Fig. 6A). Using the PfRON6-3XHA-GFP line, the expected-size HA-tagged RON6 was detected, which was efficiently depleted four days post-rapamycin treatment in blood stages (Fig. 6B).

To investigate the Pf34-PfRON6 interaction in vivo, co-immunoprecipitation studies were performed using PfRON6-3XHA-GFP blood-stage parasites and anti-HA agarose beads. Unfortunately, despite multiple attempts, confirmation or refutation of the Pf34-PfRON6 interaction was not possible, due to unexpected but persistent cross-reactivity between the anti-HA antibody and the untagged Pf34 protein.

## Discussion and conclusions

Invasion of hepatocytes by *P. falciparum* sporozoites is a bottleneck in the malaria parasite lifecycle. Inhibition of this process, particularly by vaccine-induced antibodies, is a major focus in efforts to develop means of malaria prevention. This effort is hindered by limited knowledge of the host-parasite interactions involved in hepatocyte invasion. This study has, therefore, sought to improve understanding in this area.

The approach used in this study, expressing human and *P. falciparum* proteins in a human cell line and testing them for interaction using a modified AVEXIS assay, was designed to provide the best sensitivity possible in a broad, high-throughput screen. The selection of proteins for this study included the majority of single-transmembrane sporozoite and hepatocyte surface proteins for which there is currently convincing proteomic evidence of expression. Transient mammalian cell expression can achieve post-translational modifications similar to those found in *P. falciparum* proteins. The AVEXIS assay has a strong track record in detection of biologically important interactions, including those involving *P. falciparum* proteins, even when they are strikingly weak [15–17].

Nonetheless, the approach does have limitations. AVEXIS is limited to protein ectodomains and so cannot interrogate many multi-transmembrane proteins (a substantial proportion of the hepatocyte surface proteome). Some proteins could not be expressed at all, and a further proportion were either expressed weakly or with significant degradation (Additional file 1: Figs. S1–S2). Given the high throughput nature of the screen, certainty regarding the conformational accuracy of individual proteins and the accessibility of potential interaction sites in the constructs cannot be guaranteed. It is likely that relatively weak expression is to some extent an indicator of problematic folding. Thus, although results from ‘test set’ of interactions (Fig. 1C–F) suggest that even very low bait and prey concentrations would in many cases be adequate to detect an interaction, negative results obtained with weakly-expressed proteins are of uncertain reliability.

No evidence of interactions was found with sporozoite proteins of the previously reported host receptors for sporozoite invasion (CD81, SR-BI, EphA2), nor of interactions with host proteins of the suspected sporozoite invasion ligands P36 and P52. The role of these proteins in the invasion process remains incompletely understood.

The key novel findings in this study include the interactions of Pf34 with the host protein FGFR4 and PIESP15 with LDLR. The interaction of Pf34 with FGFR4 is of very low affinity, but in the context of apposed membranes weak monovalent interactions can sum to provide significant avidity and to become biologically critical (as illustrated by the Juno-Izumo interaction which is essential for mammalian fertilization [49]). It is also possible that the Pf34—FGFR4 interaction occurs in the context of a multi-molecular complex which provides higher affinity between the host and parasite members. This possibility is supported by the fact that a co-receptor, β-klotho, contributes substantially to the binding affinity of FGFR4’s endogenous ligands [64]; unfortunately, sufficient quantities of β-klotho could not be expressed to further explore this possibility. However, an interaction between Pf34 and a second parasite protein, PfRON6, was detected. This finding is consistent with a previous study showing that PfRON6 and Pf34 co-localize in the rhoptry neck [60].

Unfortunately, the efforts to further characterize the function of these proteins proved challenging. Conditional genetic disruption confirmed the previously-reported essentiality of PfRON6 in the blood stage, but was unsuccessful for Pf34. The role of Pf34 and PfRON6 in sexual and sporozoite stages requires characterization, which may be assisted by the PfRON6 mutant parasites produced in the current study. Antibodies against Pf34 appeared to have an effect against sporozoites in vitro, which was not seen in blood-stage GIA. It is hoped that future work will clarify whether Pf34, PfRON6 and FGFR4 contribute to a functionally-important invasion complex. If so this may be specific to the *P. falciparum* sporozoite stage, given FGFR4’s liver-specific expression and the lack of interaction of murine FGFR4 with rodent malaria Pf34 orthologs [54–56]. Pf34 and PfRON6 may play important and distinct roles in sporozoite and merozoite lifecycle stages of the parasite.

### Supplementary Information

**Additional file 1: Figure S1.** Sporozoite candidate selection strategy and western blots of sporozoite protein bait library. Flow chart explaining the candidate selection process for sporozoite AVEXIS library. Western blots were performed after concentration adjustment by dilution or spin-filter concentration, with detection using anti-Ctag antibody. As described in the text and Supplementary Table 1, expression of 45 of these proteins from related constructs has previously been reported and, in these cases, this figure is intended to demonstrate quality rather than imply novelty. The band seen at c. 55 kDa in blots of pre-concentrated baits is believed to represent reactivity of the anti-Ctag antibody with a HEK293-cell protein (rather than degraded bait) as it was also seen in supernatant from cells transfected with irrelevant constructs. **Figure S2.** Hepatocyte candidate selection strategy and western blots of hepatocyte surface protein prey library. Flow chart explaining the candidate selection process for hepatocyte AVEXIS library. Proteins are arranged approximately in decreasing order of concentration as quantified by luminescence measurement. Western blots were performed after concentration adjustment by dilution or spin-filter concentration, with detection using anti-FLAG-tag antibody. As described in the text and Supplementary Table 1, expression of 127 of these proteins from related constructs has previously been reported and, in these cases, this figure is intended to demonstrate quality rather than imply novelty. **Figure S3.** (a) Murine FGFR4 does not interact with Pb34 or Py34. Absence of AVEXIS-detectable interaction of *P. berghei *and *P. yoelii* orthologs of Pf34 (‘Pb34’ [PBANKA_0721800] and ‘Py34’ [PY17X_0721800], in bait format) with murine FGFR4 (mFGFR4, in prey format). (b) Quality of purified proteins used for SPR. Purified monomeric Pf34-CD4-Bio-His (~62 kDa) and FGFR4-CD4-Bio-His protein (~64 kDa) used for SPR, on SDS-PAGE gel stained with Coomassie Blue. (c) Quality of PfRON6 C-terminal fragment bait on SDS-PAGE gel (a) and western blot (b) – no purification of this protein was undertaken, as it was captured specifically on the flow cell by anti-Fc antibody. Legend indicates expected molecular weight of each construct in kDa, including tags but excluding post-translational modifications. **Figure S4.** Kinetics of Pf34-FGFR4 and Pf34-PfRON6 interactions. (a) and (b) show SPR sensorgrams during application of FGFR4 analyte onto PfRH5 (reference, Fc1) and Pf34 (active, Fc2) coated flow cells respectively, in the experiment shown in Fig. 3C, D. (c) – (e) show Fc1, Fc2, and double-reference-subtracted curves (Fc2-1 after subtraction of Fc2-1 with buffer-only analyte) in a replicate SPR assay. (f) and (g) show SPR sensorgrams during application of Pf34 analyte onto PfRH5 (reference, Fc1) and PfRON6 C-terminal fragment (active, Fc2) coated flow cells respectively, in the experiment shown in Fig. 4C. (h) tabulates results of 1:1 binding model fitting to SPR kinetic measurement of Pf34-FGFR4 and Pf34-PfRON6 intercations. The equilibrium dissociation constant, *K*_D_, is expressed in both molar units and micrograms per millilitre. Half-life (t_1/2_) on seconds is calculated using formula 0.693/k_d_. **Figure S5.** Effect of antibodies on NF54 sporozoite invasion and 3D7 blood-stage growth. (a) – (b) shows the effect of anti-FGFR4 antibodies in ISI using NF54 parasite line (graph details are similar to Fig 5 Panel (d) – (f)). The experiment shown in panel A and B was performed in singlicate. Separate red and black lines in panel B represent control and active antibodies respectively form two independent rabbits. (c) Blood stage growth inhibitory activity of anti-Pf34 antibodies. Anti-Pf34 rabbit polyclonal IgG were tested in vitro GIA at a range of concentrations against 3D7 clone *P. falciparum. * Error bars indicated the interwell SEM triplicate wells and separate lines represent IgG from independent rabbit serum samples. **Figure S6.** Attempted generation of Pf34-disrupted parasites. (a) Schematic diagram illustrating the strategy used for attempted conditional silencing of Pf34 using CRISPR/Cas9 system. Parasite genomic locus is shown with Pf34 CDS, 5’ and 3’ UTRs and location of primers are indicated with arrows. Repair plasmid is shown with homology regions (HR1 and HR2), re-codonized Pf34 (RC-Pf34) with the insertion of loxP sites (see materials and methods). Modified locus is shown if the integration of repair plasmid into NF54-DiCre parasite genome occurs following transfection and after rapamycin treatment. (b) Strategy used for attempted generation of Pf34-3XHA-GFP cKO parasites. A similar repair plasmid that is shown in Fig 6A with the addition of 3XHA after signal peptide, and eGFP after silent loxP cassette was used for transfection. No drug resistant parasites were observed for Pf34 cKO and Pf34-3XHA-GFP cKO parasites. **Figure S7**. Generation of PfRON6-disrupted parasites. (a) Generation of PfRON6 cKO parasites. Parasite genomic locus is shown with PfRON6 CDS, 5’ and 3’ UTRs and location of primers are indicated with arrows. Repair plasmid is shown with homology regions (HR1 and HR2), re-codonized PfRON6 (RC-PfRON6) with the insertion of loxP sites (see Methods). Modified locus is shown after the integration of repair plasmid into NF54-DiCre parasite genome following transfection and after rapamycin treatment. Diagnostic PCR using the primer sets P1/P5 and P6/P4 (805 bp and 719 bp respectively) is showing the integration of PfRON6 cKO repair plasmid and the absence of wild type contamination is shown using primer sets P1/P2 and P3/P4 in PfRON6 cKO clone B4 and F7 (that produce 864 bp and 636 bp amplicons respectively using wild type genomic DNA). (b) Generation of PfRON6-3XHA-GFP cKO parasites. A similar repair plasmid that is shown in Fig 6C with the addition of 3XHA and GFP was used for transfection. Diagnostic PCRs are shown for the correct integration of PfRON6-3XHA-GFP repair plasmid in the endogenous locus using the primer sets P1/P5 and P6/P4 (805 bp and 1541 bp, respectively) and the absence of non-transfectants in PfRON6-3XHA-GFP cKO clone B3 and E10 using primer sets P1/P2 and P3/P4 as above.

**Additional file 2: Table S1.** *S*porozoite protein ectodomain library details. Details include bait index number (corresponding to numbering in Fig. 3A), construct boundaries and sequence, and expression levels (corresponding to Fig. 2A). Candidates excluded from the study are listed in the table.

**Additional file 3: Table S2.** Human hepatocyte protein ectodomain library details. Details include construct boundaries and sequence, and expression levels (corresponding to Fig. 2B). Integrin α and β chain constructs are listed on the second worksheet, and the results of integrin heterodimer expression are listed on the third worksheet. Candidates excluded from the study are listed in the table.

**Additional file 4: Table S3.** First worksheet presents complete AVEXIS screen results, presented in terms of double-corrected signal:noise ratio (see Methods). Color scale denotes the signal:noise ratio of each interaction, ranging from dark green (low) through yellow to dark red (high). Second and third worksheets show results with FGFR4 prey against all sporozoite baits (as shown in Fig. 3A) and with Pf34 prey against all sporozoite baits (as shown in Fig. 4B).

**Additional file 5: Table S4.** All protein pairs with a signal:noise ratio exceeding 5 in the initial AVEXIS screen.

**Additional file 6: Table S5.** List of oligonucleotides used in this study.

## Data Availability

All data sets generated or analysed during this study are included in this manuscript (and in its Additional files).
