# Supplementary material for: A screen for Plasmodium falciparum sporozoite surface protein binding to human hepatocyte surface receptors identifies novel host–pathogen interactions
Source: Malar J. 2024 May 16;23:151. doi: 10.1186/s12936-024-04913-2 (PMC11098746; doi:10.1186/s12936-024-04913-2)

Supplementary Figure 1

Sporozoite candidates selection

1. No signal peptide or multiple transmembrane domains (n=32).
2. Functional evidence of other roles e.g. metabolic enzyme, ER chaperone etc (n=45)
3. Proteins lacking convincing evidence of sporozoite surface expression (n=29)

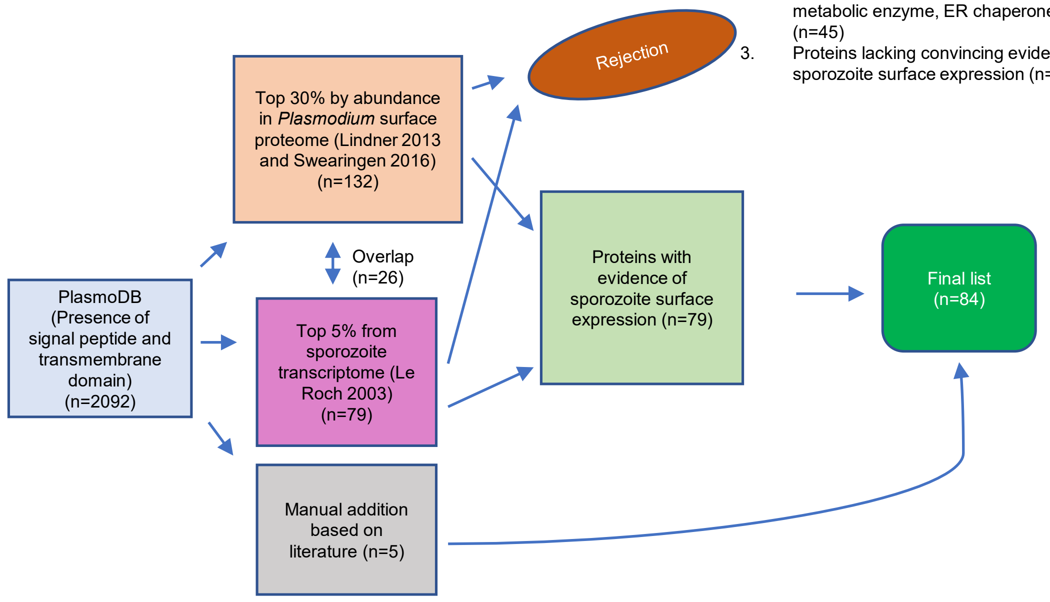

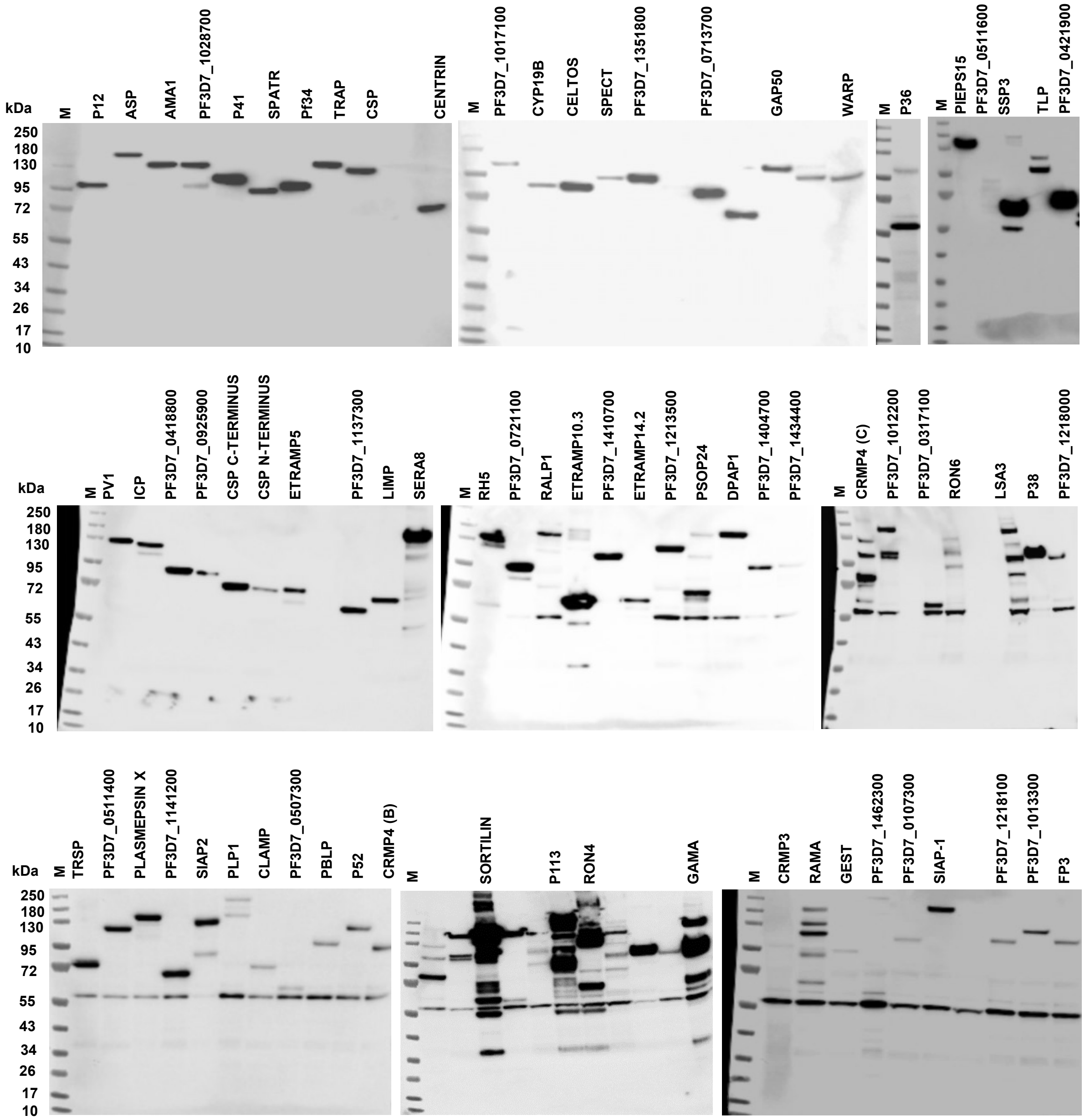

Supplementary Figure 2

Hepatocyte candidates selection

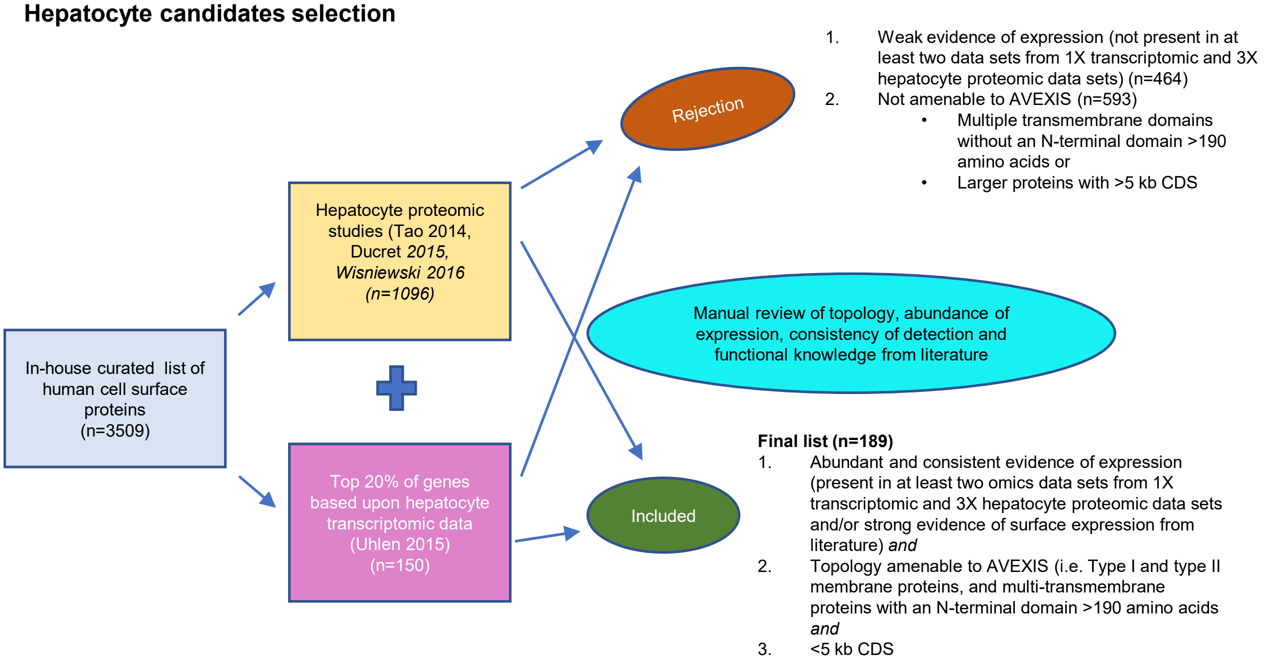

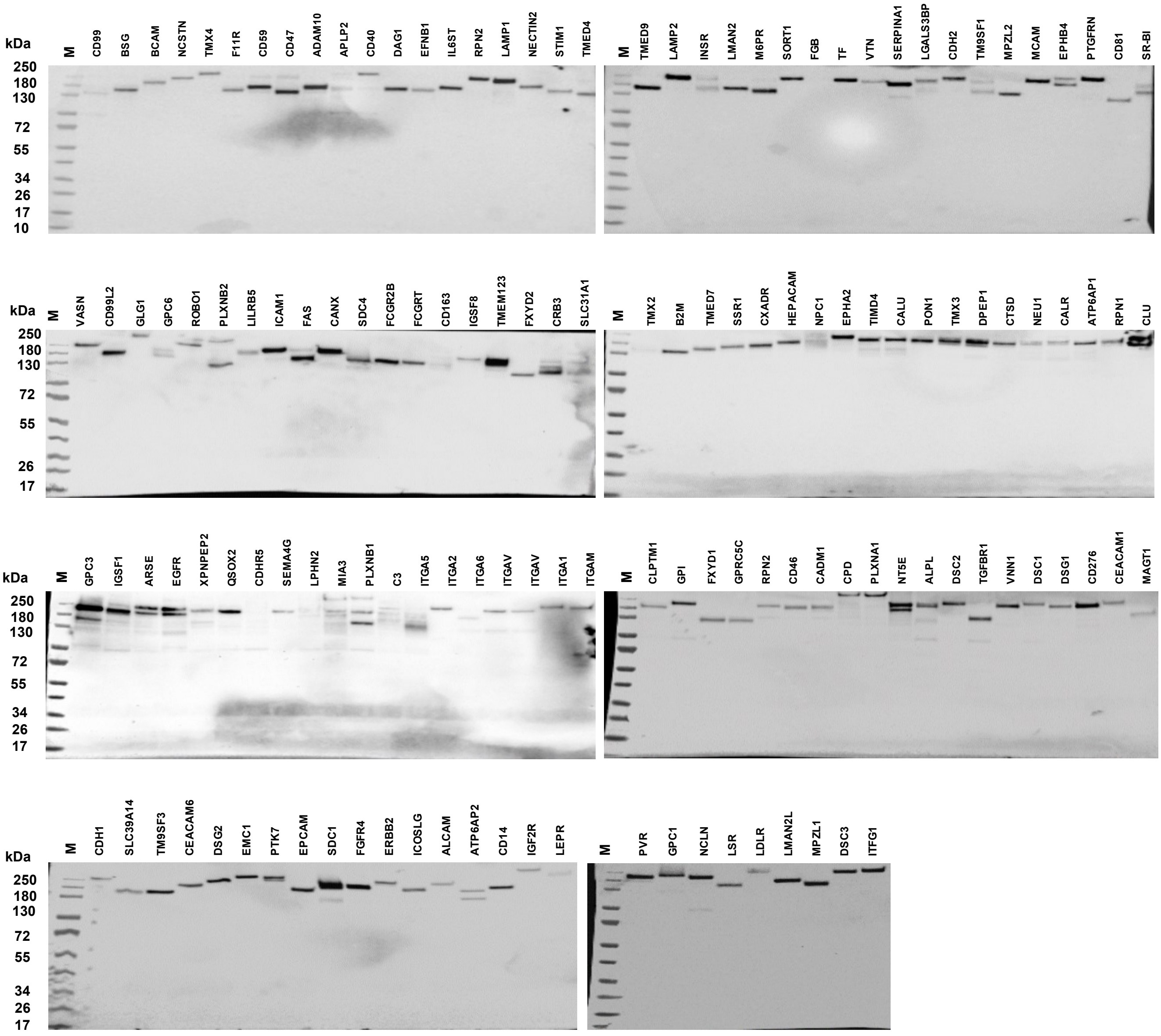

Supplementary Figure 3

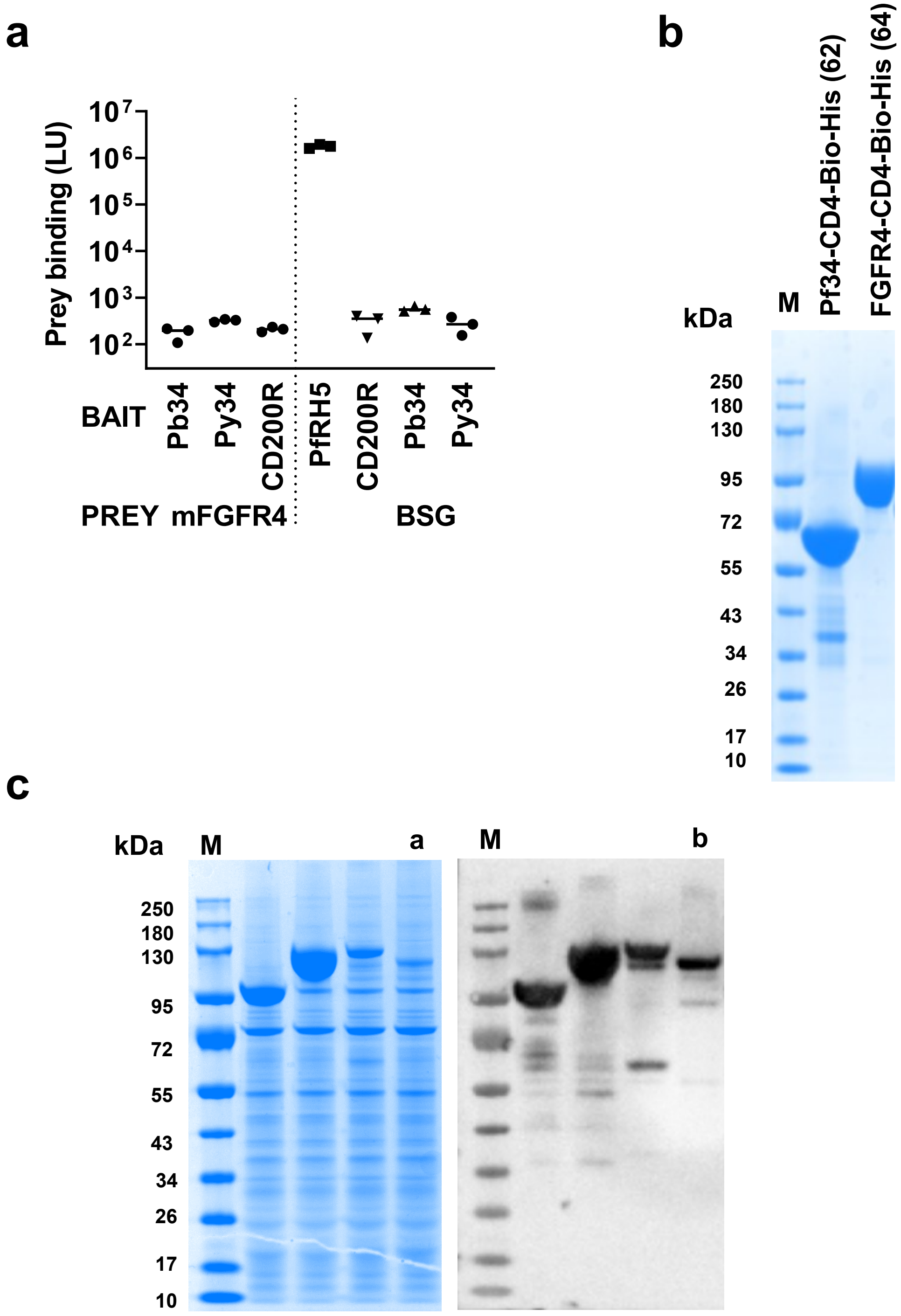

a, b: PfRON6 C-terminal bait (100)

Supplementary Figure 4

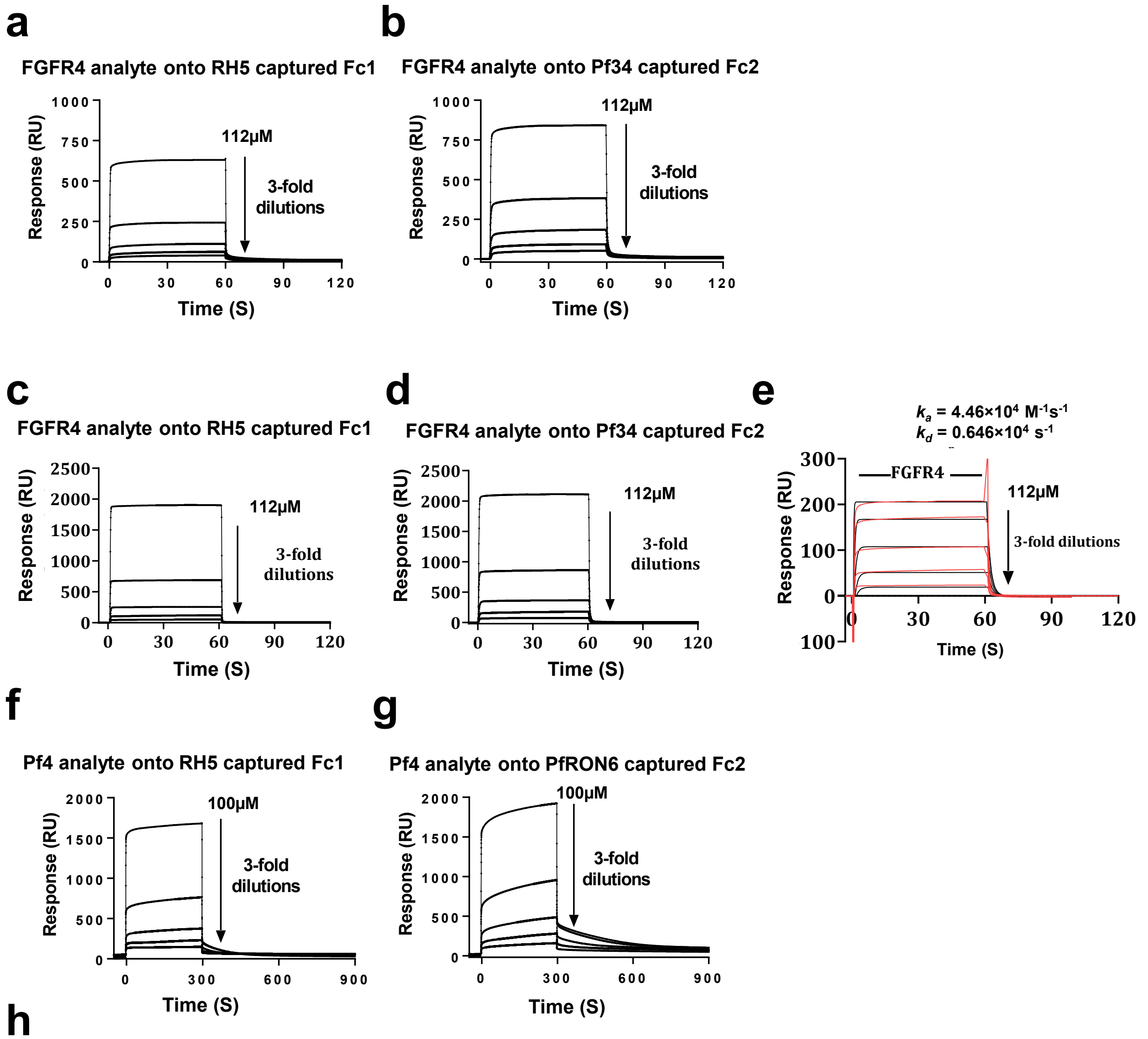

h

Interaction	Experiment	Ligand	Analyte	Ka ($M^{-1}S^{-1}$)	SE of Ka ($M^{-1}S^{-1}$)	Kd (S^{-1})	SE of Kd (S^{-1})	K_D (M)	K_D (mg/mL)	Interaction half-life (sec)	R_{max} (RU)	Chi^2 (RU^2)
**Pf34-FGFR4**	1	Pf34	FGFR4	1.6E+04	1.8E+02	6.8E-01	6.7E-03	4.7E-05			288.9	27.5
	2	Pf34	FRGR4	4.4E+04	8.4E+02	6.4E-01	1.1E-02	1.4E-05			232	120
	**Median**			**3.0E+04**	**5.1E+02**	**6.6E-01**	**8.9E-03**	**3.1E-05**	**2**	**1**		
**Pf34-PfRON6**	1	PfRON6 C-terminal fragment	Pf34	5.2E+02	2.2E+00	1.5E-03	5.0E+06	2.9E-06			196	293

Supplementary Figure 5

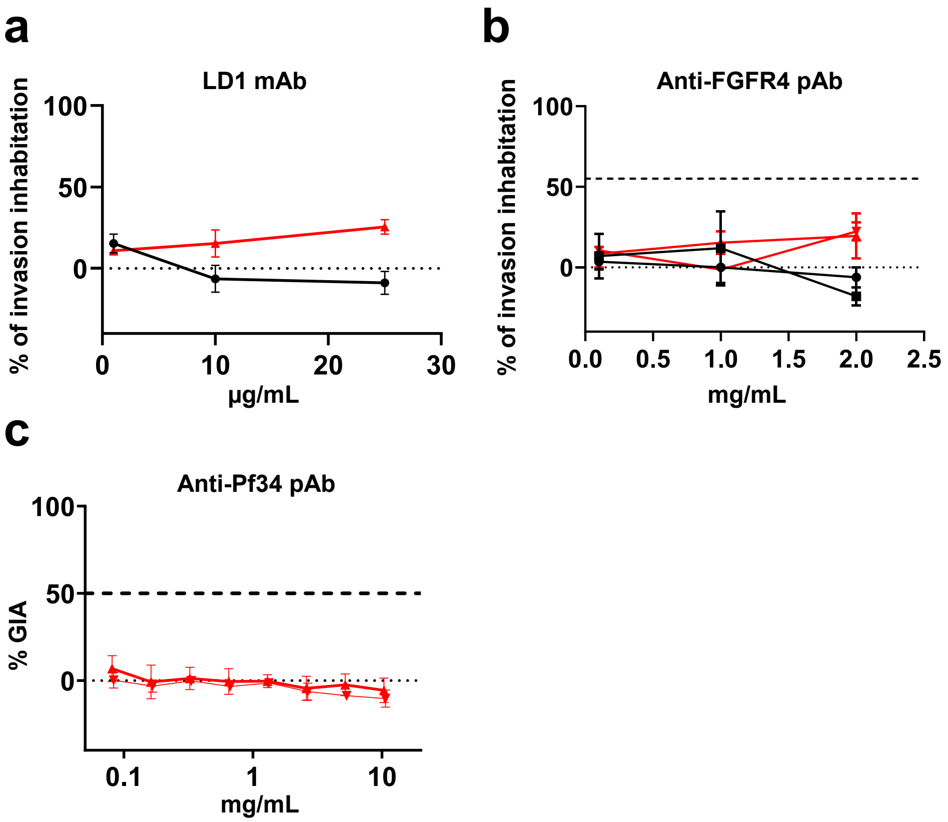

Supplementary Figure 6

a

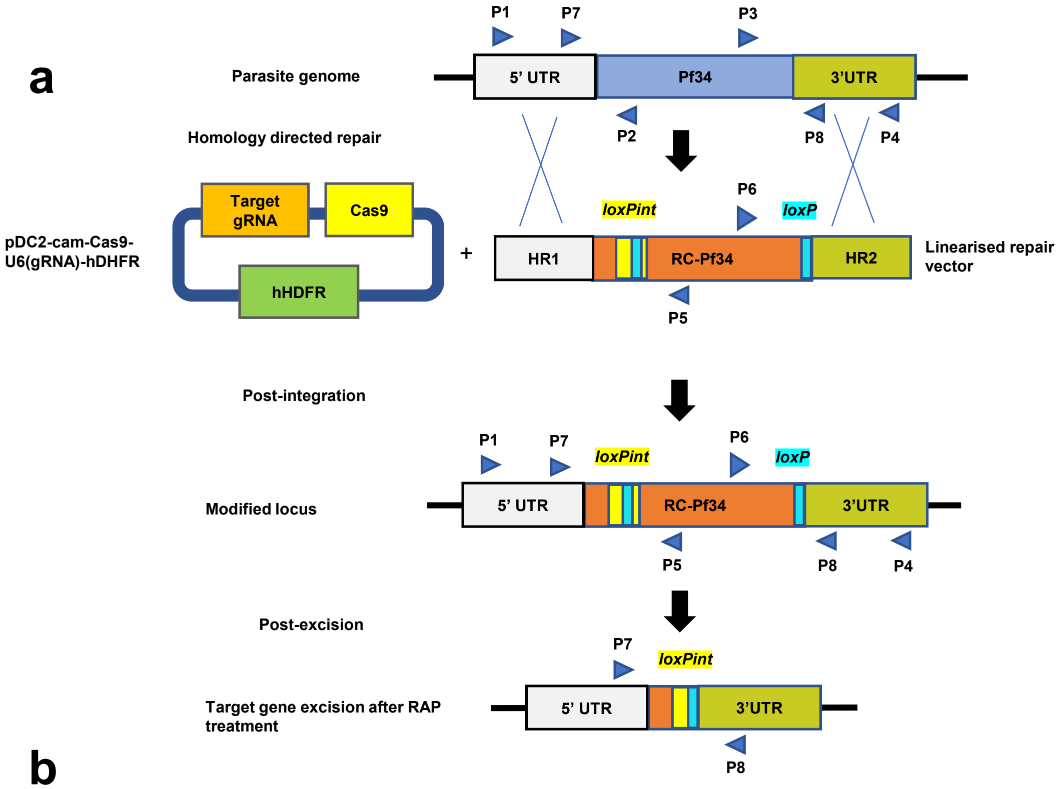

b

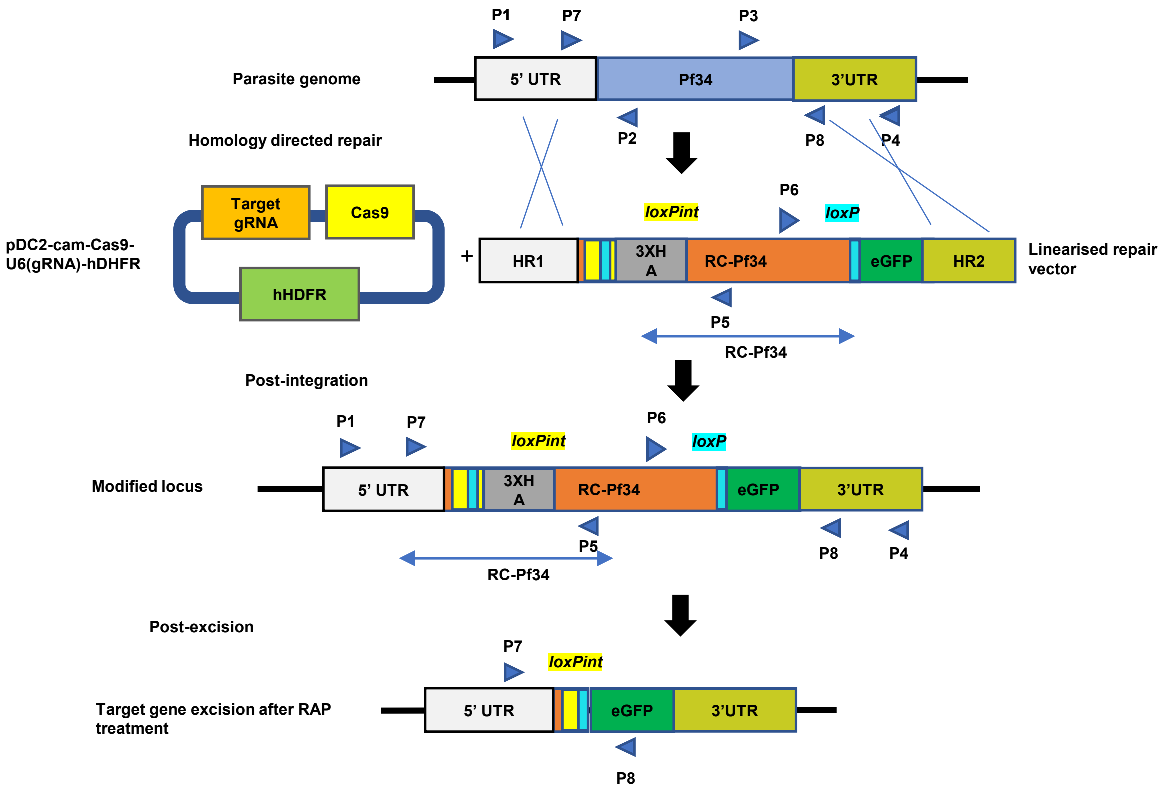

Supplementary Figure 7

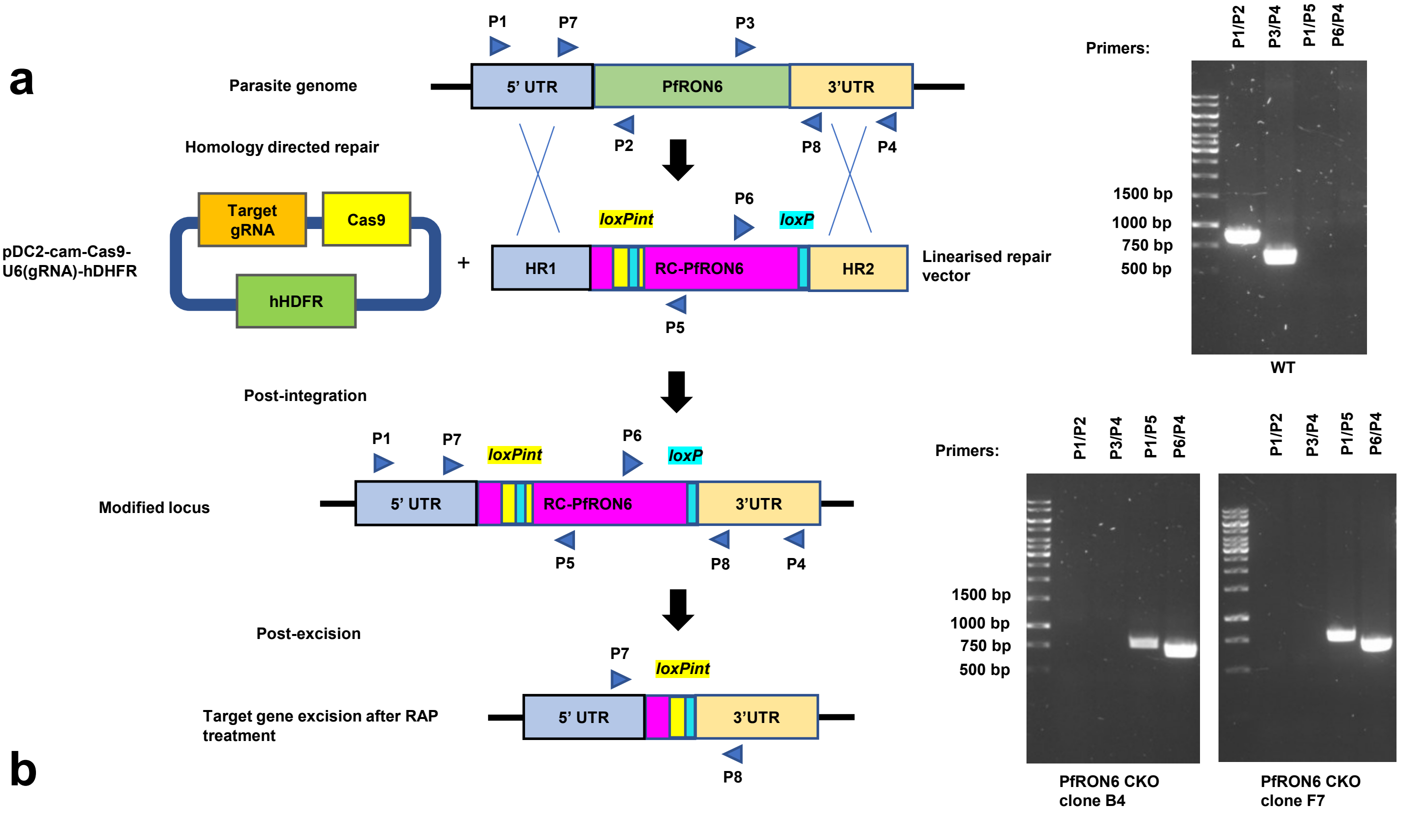

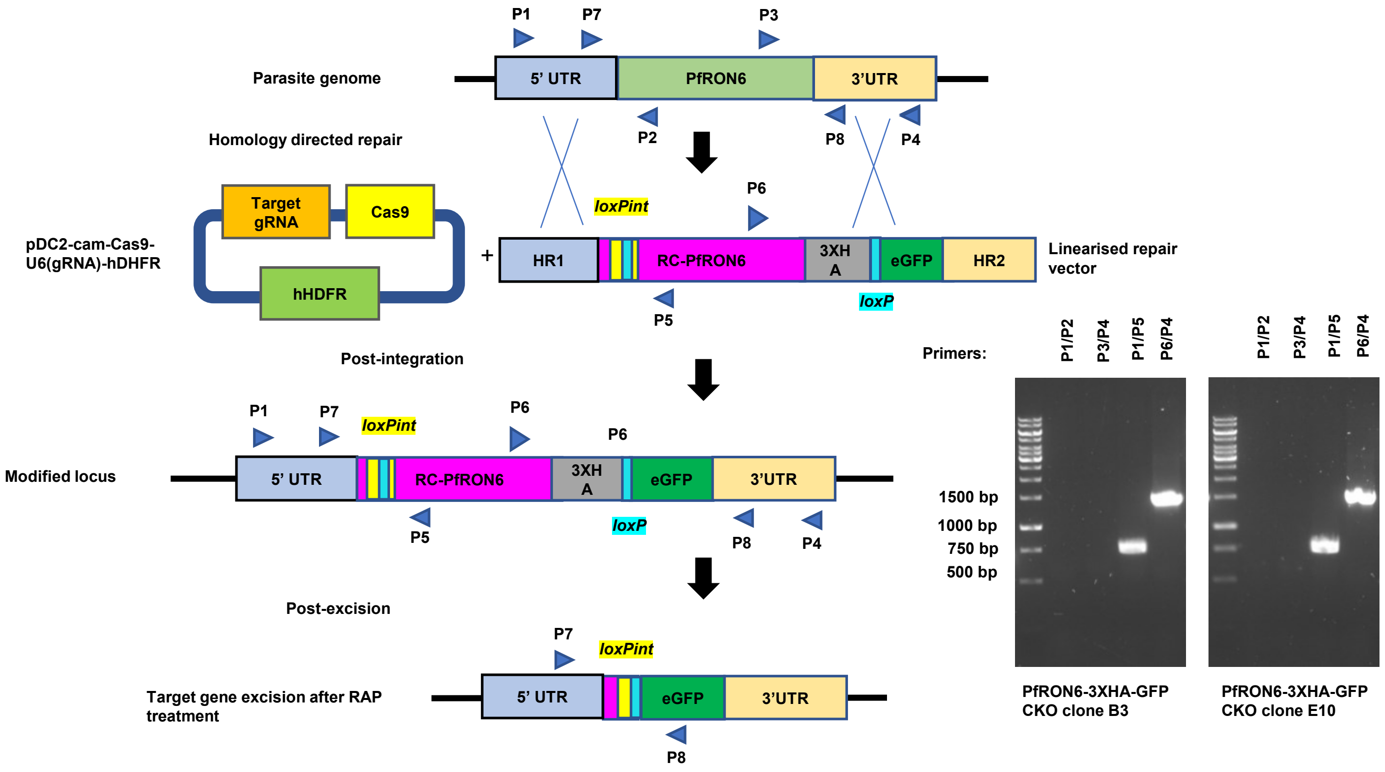

Supplement: Supplementary file 1 — Additional file 1: Figure S1. Sporozoite candidate selection strategy and western blots of sporozoite protein bait library. Flow chart explaining the candidate selection process for sporozoite AVEXIS library. Western blots were performed after concentration adjustment by dilution or spin-filter concentration, with detection using anti-Ctag antibody. As described in the text and Supplementary Table 1, expression of 45 of these proteins from related constructs has previously been reported and, in these cases, this figure is intended to demonstrate quality rather than imply novelty. The band seen at c. 55 kDa in blots of pre-concentrated baits is believed to represent reactivity of the anti-Ctag antibody with a HEK293-cell protein (rather than degraded bait) as it was also seen in supernatant from cells transfected with irrelevant constructs. Figure S2. Hepatocyte candidate selection strategy and western blots of hepatocyte surface protein prey library. Flow chart explaining the candidate selection process for hepatocyte AVEXIS library. Proteins are arranged approximately in decreasing order of concentration as quantified by luminescence measurement. Western blots were performed after concentration adjustment by dilution or spin-filter concentration, with detection using anti-FLAG-tag antibody. As described in the text and Supplementary Table 1, expression of 127 of these proteins from related constructs has previously been reported and, in these cases, this figure is intended to demonstrate quality rather than imply novelty. Figure S3. (a) Murine FGFR4 does not interact with Pb34 or Py34. Absence of AVEXIS-detectable interaction of P. berghei and P. yoelii orthologs of Pf34 (‘Pb34’ [PBANKA_0721800] and ‘Py34’ [PY17X_0721800], in bait format) with murine FGFR4 (mFGFR4, in prey format). (b) Quality of purified proteins used for SPR. Purified monomeric Pf34-CD4-Bio-His (~62 kDa) and FGFR4-CD4-Bio-His protein (~64 kDa) used for SPR, on SDS-PAGE gel stained with Coomassie [file 12936_2024_4913_MOESM1_ESM.pdf]
